# Contrasting Effects of Tagging Turnip Mosaic Virus Proteins

**DOI:** 10.3390/pathogens15060611

**Published:** 2026-06-08

**Authors:** Amany E. Gomaa, Eric Parperides, Xin-Qiu Yao, Gabriela Espinoza Vergara, Ziomara Jurado, Hernan Garcia-Ruiz

**Affiliations:** 1 Department of Plant Pathology and Nebraska Center for Virology, University of Nebraska-Lincoln, Lincoln, NE 68583, USA; agomaa2@huskers.unl.edu (A.E.G.);; 2 Department of Botany, Faculty of Science, Mansoura University, Mansoura 35516, Egypt; 3 Department of Chemistry, University of Nebraska-Omaha, Omaha, NE 68182, USA; 4 Departamento de Parasitología Agrícola, Universidad Autónoma Chapingo, Texcoco 56230, Mexico; gabyespver@gmail.com; 5 Biology Department, Nebraska Wesleyan University, Lincoln, NE 68504, USA

**Keywords:** potyvirus, epitope tagging, local and systemic infection, infectious clone, nuclear inclusion protein b, temperature-sensitive infection, AlphaFold

## Abstract

*Potyvirus rapae* (turnip mosaic virus, TuMV) is widely used as a model system in plant–virus interaction studies. The TuMV RNA genome encodes 11 proteins, some of which remain poorly characterized, while the functions of others are well defined. Studying individual proteins in isolation may not recapitulate native expression levels, subcellular localization, and interaction with host factors during virus replication and movement. An alternative approach is to tag individual viral proteins in the context of an infectious clone. Epitope tags may alter protein functions and affect viral replication, movement, or a combination of essential steps, thus leading to changes in pathogenicity. Because they have central roles in viral infection, here we measured the effect of individually tagging the helper component proteinase (HC-Pro) and nuclear inclusion protein b (NIb) with a 6His-3xFLAG tag. Epitope tags were placed at the N-terminus of HC-Pro and the N- and C-termini of NIb within a TuMV infectious clone carrying coding sequences for the green fluorescent protein (TuMV-GFP). Constructs carrying a tagged HC-Pro displayed pathogenicity similar to that observed for TuMV-GFP in *Nicotiana benthamiana* and *Arabidopsis thaliana* plants. In contrast, infectivity of NIb-tagged clones became temperature sensitive and, even at the permissive temperature, showed reduced pathogenicity compared to TuMV-GFP. Providing a silencing suppressor in trans did not restore infection efficiency, suggesting reduced viral fitness due to structural or functional disruption caused by the epitope tags. Structural models generated using AlphaFold2 showed no effect of the tag on HC-Pro. In contrast, structural models illustrated tag interference with the NIb catalytic site. AlphaFold2 was further used to predict the structural impact of several tags on NIb and to predict the effect of a 6HIS-3xFlag tag on all other TuMV proteins. This study provides a broadly applicable framework for selecting suitable epitope tags to mark viral proteins and maintain function in the context of virus infection.

## 1. Introduction

Potyviruses (family *Potyviridae*, genus *Potyvirus*) are the largest group of plant RNA viruses, infecting a wide range of economically important crops and causing substantial agricultural losses worldwide [1]. Potyviruses rely on a single open reading frame (ORF) to produce eleven proteins, each of which has evolved to execute specialized functions during replication, movement, or inactivation of plant antiviral responses, all in cooperation with host factors [1,2]. *Potyvirus rapae* (turnip mosaic virus, TuMV) is a member of the genus *Potyvirus* [3], and its genome consists of one positive-sense single-stranded RNA with a length of about 10 kb that is translated into a single large polyprotein, that is subsequently processed into ten mature viral proteins by the coordinated action of three virus-encoded proteases [1]. Additionally, P3-PIPO is produced by transcriptional slippage by the viral RNA-dependent RNA polymerase [2]. The functions of some potyviral proteins are well defined and others remain poorly characterized. However, further investigation is needed to better identify critical host factors interacting with viral proteins during the infection cycle [2,4]. A powerful tool for identifying host factors is the use of viral infectious clones, which are full-length DNA copies of viral genomes (DNA or cDNA for RNA viruses) placed under control of a promoter and cloned into bacterial binary plasmids [5]. These constructs allow genetic manipulation of viral genomes, mutational analysis, and have been used to determine viral gene functions, gene expression, mechanisms of virus replication, movement, and encapsidation, as well as fundamental mechanisms of plant–virus interaction [6]. For RNA viruses, cDNA clones can also be modified and used as vectors for virus-induced gene silencing (VIGS) and used to study plant gene function, produce proteins, or for plant gene editing. The first infectious cDNA clone was generated to express the brome mosaic virus (BMV) [7]. After that, the same approach has been used to generate viral cDNA clones for a growing number of viruses [5]. Additionally, adding a visual marker, such as a green fluorescent protein (GFP) or its variants (YFP, CFP, mCherry), enables real-time visualization of virus infection, cell-to-cell and systemic movement within host tissues [8].

To identify protein–protein interactions, and to dissect virus–host interactions, epitope tagging of viral proteins has become an indispensable strategy [9,10]. Epitope tags are short nucleotide sequences coding for a few amino acids fused to proteins of interest to facilitate detection, visualization, purification, quantification, functional analysis, or identification of interaction partners [11,12]. These tags are broadly classified based on their purpose [13]. Epitope tags, such as human influenza virus hemagglutinin protein (HA), FLAG, or the human c-Myc (Myc) protein, are genetically fused to proteins of interest and can be recognized with high specificity by commercial antibodies [12]. These tags facilitate sensitive immunodetection by methods, such as enzyme-linked immunosorbent assay (ELISA), western blotting, immunofluorescence, and immunoprecipitation, and are powerful experimental tools, especially when antibodies against the native viral proteins are not available [13,14]. Affinity tags, such as the polyhistidine (His), glutathione S-transferase (GST), and Strep-tag, provide strong, selective binding to immobilized substrates and are primarily employed for the efficient purification of recombinant proteins via antibody-mediated binding [15]. Fluorescent tags permit live imaging of protein dynamics, while advanced approaches, such as proximity labeling (e.g., TurboID), enable identification of transient or spatially restricted protein associations in living cells [2,16].

Collectively, protein tags enable detection, tracking, and isolation of viral proteins for structural and biochemical assays and support downstream analyses such as mass spectrometry and in vitro interaction studies [8,10]. Importantly, when protein tags are used to investigate virus–plant protein interactions, a replication-competent infectious clone carrying the full-length viral genome is essential to preserve the timing of protein expression, relative abundance, and subcellular localization, and to closely mimic wild-type infection dynamics [17,18]. Tagging and expressing viral proteins in the context of the entire virus provides a significant advantage over expressing tagged proteins individually, as in the latter approach, virus replication and movement do not occur [12,19].

Although protein tagging is a powerful strategy in molecular biology, several challenges must be considered. A primary concern is that the addition of a tag can alter the native structure, stability, or functions of the protein. Tags may interfere with protein folding, enzymatic activity, or interaction surfaces, particularly when fused to proteins with compact structures or critical terminal domains [10]. The position of the tag (N- or C-terminus) can also affect protein function [20]. Tagging viral proteins presents additional complexity because viral genomes are highly compact and functionally constrained. Inserting tags can disrupt overlapping reading frames, regulatory elements, or replication signals, potentially impacting viral replication, movement, or symptom development [18,21]. As a result, a virus carrying tagged proteins may no longer mimic wild-type infection dynamics.

Another challenge is the size of the tag. Large tags, such as GFP or GST, can sterically hinder protein interactions or prevent correct subcellular localization [22]. Additionally, even small epitope tags may interfere with interaction domains [23]. Furthermore, for affinity and epitope tags, antibody specificity and background binding may produce false positives in immunodetection assays [24]. Similarly, in proximity labeling approaches (e.g., TurboID), the labeling radius may result in the identification of neighboring proteins that are not true interactors, requiring careful experimental controls and validation [25].

In potyviruses, N-terminal motifs of HC-Pro mediate aphid transmission, the central F(Y)RNK motif influences symptom severity, genome amplification, movement, and RNA silencing suppression, and the C-terminal cysteine protease domain processes the viral polyprotein [26,27]. As a viral suppressor of RNA silencing, HC-Pro binds and sequesters a subset of cellular and virus-derived short-interfering RNAs, disrupts Argonaute complexes, and interacts with host factors to counter antiviral defenses [12,27]. HC-Pro also engages with host proteins, such as the 20S proteasome, translation factors, chloroplast proteins, and enzymes in metabolic pathways, affecting replication, movement, symptom development, and host physiology [26,28,29,30].

NIb is the viral RNA-dependent RNA polymerase (RdRp) essential for genome replication of potyviruses and is structurally conserved within superfamily II RdRps, containing the characteristic palm, thumb, and fingers subdomains and the catalytic GDD motif required for polymerase activity [1,31,32]. Beyond its replication function, NIb acts as a nucleocytoplasmic shuttling protein containing functional nuclear localization signals and nuclear export signals. Mutations in localization signal motifs significantly reduced viral RNA accumulation and systemic infection, indicating that proper nucleocytoplasmic trafficking of NIb is required for efficient viral infection [33]. NIb is also one of the most interaction-rich potyviral proteins, forming complexes with other viral components, such as viral genome-linked protein (VPg), coat protein (CP), and genomic RNA, while simultaneously interacting with numerous host factors involved in translation, RNA metabolism, protein folding, SUMOylation, and autophagy [31].

These extensive interactions and biological functions position both HC-Pro and NIb at the interface of viral replication and host cellular pathways, and make these viral proteins ideal candidates for epitope tagging with the end goal of identifying host-interacting factors and molecular networks and understanding how potyviruses hijack host machinery for replication, movement and suppression of antiviral defense. Accordingly, in this study, we focused on HC-Pro and NIb, made infectious clones expressing tagged HC-Pro or Nib, and tested the infection efficiency and pathogenicity of full-length TuMV infectious clones expressing HC-Pro or NIb tagged at the N- or C-terminus. Additionally, we used AlphaFold2 to model protein structure and predict protein functionality after the addition of tags of interest and used it as a tool for selection of epitope tags that minimized structural and functional disruption of the viral proteins. AlphaFold2 models showed that 6HIS-3xFLAG changed the structure of NIb and partially blocked the catalytic GDD motif. Infectivity assays showed that tagging NIb resulted in temperature sensitivity, reduced infection efficiency, and a delay in the progression of systemic infection. In contrast, the same tag did not change the structure and did not affect the biological activity of HC-Pro. AlphaFold2 models were generated for all TuMV proteins and for several tags. Models make predictions about tags interfering or not with the protein function. The approach developed here establishes a platform for selecting protein tags predicted to maintain protein function in the context of virus infection and is a powerful tool for co-immunoprecipitation assays in a biologically relevant context.

## 2. Materials and Methods

### 2.1. Plasmid Constructions

**Figure 1:**
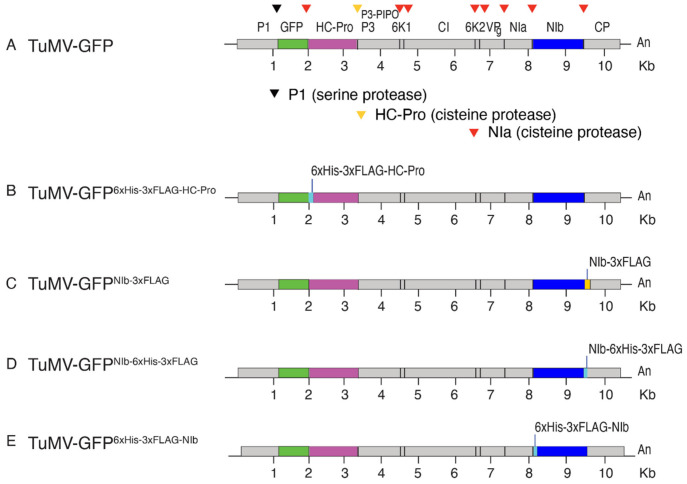
Schematic representation of TuMV clones used in this study and based on pCB302 plasmid [6]. Arrows point to polyprotein cleavage sites by proteinases P1, HC-Pro, and NIa. (**A**) Plasmid carrying TuMV with GFP between P1 and HC-Pro. This clone was used for the construction of all derivatives. (**B**) HC-Pro is tagged at the 5′-terminus with 6xHis-3xFLAG. (**C**) NIb is tagged by 3xFLAG at the 3′ end. (**D**) NIb is tagged with 6xHis-3xFLAG at the 3′ end. (**E**) NIb is tagged with 6xHis-3xFLAG at the 5′ end.

The clones used here were derived from TuMV-GFP [6] using standard cloning techniques, and destination (pCB302) vectors were used. The viral constructs are shown in Figure 1.

To tag HC-Pro at the N-terminus with 6xHis-3xFLAG, overlapping fragments were generated to add sequences to the 5′ end. Using plasmid pCB3202-TuMV-GFP [6] as a template, a PCR fragment (A) containing part of P1 and GFP was generated using primers 111 and 674. In antisense orientation, primer 674 binds to the last 12 nt of GFP (nt 1208 to 1219), adds a 6HIS-3xFLAG tag to HC-Pro, introduces an NIa cleavage site (CVYHQAG) between GFP and the 6HIS-3xFLAG on HC-pro, and adds a NheI restriction site. An overlapping PCR fragment (B) was created using plasmid pCB3202-TuMV-GFP as a template and primer 675 and primer 110. Primers 674 and 675 are overlapping. In antisense orientation, primer 110 binds to HC-Pro downstream of the AgeI site. Overlapping PCR fragments, A and B, were fused by stitching PCR and amplified by PCR using primers 110 and 111 and used as a source of insert. Both the vector (pCB3202-TuMV-GFP) and the insert (PCR product) were digested with PvuII-AgeI and ligated. Good clones were identified by NheI-EcoRI double digestion and confirmed by Sanger sequencing. The resulting plasmid is pCB3202-TuMV-GFP-6HIS3xFLAg-HC-Pro (lab plasmid 196).

To tag NIb, an intermediate cloning vector was used based on pENTR [34]. Plasmid pENTR-NIb-CP (lab number 239) was generated by PCR and TOPO cloning. Using plasmid pCB3202-TuMV-GFP as a template, a PCR fragment was generated using primers 780 and 719 and added as an insert in pENTR. Plasmid pENTR-NIb-CP contains the C-terminal half of TuMV NIb, the entire coat protein, the 3′ UTR, and part of the pCB302 vector. Plasmid pENTR-NIb-CP was used to add nucleotide sequence coding for tags fused to NIb by rolling circle amplification PCR [35] as follows. Using plasmid pENTR-NIb-CP as a template, sequence coding for 3xFLAG was fused to the NIb coding sequence, and an NIa cleavage site was added between the tag and the coat protein using overlapping oligonucleotide primers 833 and 834 by rolling circle amplification PCR. In this strategy, oligonucleotide primer 843 contains the desired modifications, including a sequence for the 3xFLAG fused to NIb and an NIa cleavage site. The parental plasmid was removed by DpnI digestion, and the resulting products were transformed into Escherichia coli DH5α competent cells. Positive clones were confirmed by sequencing. The resulting plasmid pENTR-NIb-3xFLAG-CP (lab number 257) was used as a source of insert. The insert and vector pCB3202-TuMV-GFP were digested with MluI and PvuI and ligated. Good clones were identified by HpaI-PvuI double digestion and verified by Sanger sequencing. The resulting plasmid carries sequences for a 3xFLAG tag at the 3′ part of NIb (pCB3202-TuMV-GFP-NIB-3xFLAG, lab number 348). A similar approach was used to add sequence coding for a 6HIS-3xFLAG tag at the 3′ end and 5′ end of NIb. Oligonucleotide primers 833 and 835 were used to add the 6HIS-3xFLAG tag at the 3′ end part and oligonucleotide primers 1093 and 1094 were used to add sequence coding for the 6HIS-3xFLAG tag at the 5′ end. The resulting plasmids pCB3202-TuMV-GFP-NIB-6HIS-3xFLAG (lab number 349) and pCB3202-TuMV-GFP-6His-3xFLAG-NIB (lab number 380) have sequences for the 6His-3xFLAG tag at the 3′ end and 5′ end, respectively. The destination vector pCB302 [34] was introduced into *Agrobacterium tumefaciens* by electroporation. The sequences of all oligonucleotide primers (5′ to 3′ orientation) are provided in Table S1.

### 2.2. Plant Materials and Virus Inoculation

Wild-type *Nicotiana benthamiana* plants were grown in a greenhouse and under controlled conditions (24 °C, 16 h light/8 h dark). *Arabidopsis thaliana* plants, ecotype Columbia-0, were either wild type or *dcl2-1 dcl3-1 dcl4-2* triple mutant [6]. Seeds were suspended in 0.1% agarose and vernalized at 4 °C for 48 h. Sterile soil was pre-moistened, and seeds were sown. Both *N. benthamiana* and *A. thaliana* plants were maintained at 24 °C, 16 h light/8 h dark for 16 or 21 days, respectively, before virus inoculation. After virus inoculation through agrobacterium [36] carrying a TuMV-GFP clone of interest, plants were grown at 22 °C (16 h light/8 h dark) for up to 20 days.

### 2.3. Protein Extraction and Western Blot Assay

Systematically infected leaves (0.15 g) were homogenized in 600 μL of glycine grinding buffer (0.1 M glycine-NaOH, pH 9.0, 100 mM NaCl, 10 mM EDTA, 2% sodium dodecyl sulphate, and 1% sodium lauroyl sarcosine) as described in [19,30,37], with the addition of Zirconia beads. Tissue homogenization was performed using a bead beater for 3 min, followed by centrifugation at 14,000 rpm for 3 min. From the resulting supernatant, 25 μL was mixed with 50 μL of 2X protein dissociation buffer to achieve a final concentration of 1X, then diluted to 1/8X, heated at 100 °C for 3 min, and stored at −80 °C. Total extracted proteins were separated using Mini-Protean TGX precast gels (Bio-Rad^®^) by loading 5–10 μL per lane and running for 60 min. Proteins were then transferred onto Immobilon-FL PVDF membranes (0.45 μm pore size) (Millipore Sigma, Saint Louis, MO, USA, catalog number IPFL00010) for 70 min at 100 V in transfer buffer. Membranes were stained with Ponceau S solution (Milllipore Sigma, catalog number 09276-6X1EA-F) for 10 min, imaged, and destained with 1% PBS-T. Blocking was performed with 5% non-fat milk for at least 30 min before incubation with primary antibodies. Rubisco and antibodies to detect HSP70 (Thermo Fisher Scientific, Waltham, MA, USA, catalog number AS09 592) were used as loading controls. Viral proteins HC-Pro and NIb were detected using anti-His (Cell Signaling Technology, Danvers, MA, USA, catalog number 9991S) and anti-FLAG (Milllipore Sigma, catalog number A8592-0.2 mg) antibodies following the manufacturer’s instructions. Membranes were washed three times with PBS-T (5 min each), and signals were visualized using Clarity™ Western ECL substrate (Bio-Rad^®^, Hercules, CA, USA, catalog number 1705060). Images were captured with a ChemiDoc Imaging System (Bio-Rad^®^, catalog number 170-8280).

### 2.4. AlphaFold2 Prediction Models

We used AlphaFold2 (Google DeepMind, London, UK) [38] to generate a 3D structure of HC-Pro and NIb in wild-type form and in the presence of epitope tags at the N- or C- terminus. A list of tags used is provided in Table S2. The algorithms in AlphaFold predict the protein structure based on the amino acid sequence. Highly ranked predicted models were visualized and edited using UCSF ChimeraX (https://www.cgl.ucsf.edu/chimerax/, accessed on 3 May 2025) [39]. We also used the ChimeraX Matchmaker tool to make an alignment between the wild-type structure of the protein and the structure of the protein with the tag. We also color the sequence of the tag differently from the original protein structure using the selection tool. The same approach was used to predict the effect of 6xHis-3xFALG on other TuMV proteins. Amino acid sequences with and without tags are shown in Table S4.

### 2.5. Predicting the Effect of Tags on Protein Function

Solvent-accessible surface area (SASA) changes at the active site, and surrounding residues were used to assess the potential effect of tag modification on protein function. SASA was calculated using the cpptraj program of AmberTools 23 (Amber MD software, ambermd.org) [40]. Three residue sets were analyzed, and their SASA values were measured in the presence and absence of tags from HC-Pro, NIb, and other TuMV proteins. Active-site (AS) residues were identified (Table 1). Residues proximal to the active site (AS_5A) were defined as those with a minimum non-hydrogen atomic distance of ≤5 Å from any active-site residue. Residues lining the pore that passes through the active site (putative substrate-binding pathway, denoted by “Pore Lining”) were identified using MOLEonlineversion 2.5 (Elixir, CZ, Prague, Czech Republic; moleonline.cz) [41]. Percent differences in SASA were calculated for each residue set using the untagged protein as the reference. Similar analyses were performed for other potyviral proteins. Data analysis was performed using an in-house R script (version 4.6.0) provided as Supplemental Materials (S6: run_sasa.r. S7: funs.R).

## 3. Results

### 3.1. Tagging HC-Pro with 6His-3xFlag Did Not Affect Local nor Systemic Infection

**Figure 2:**
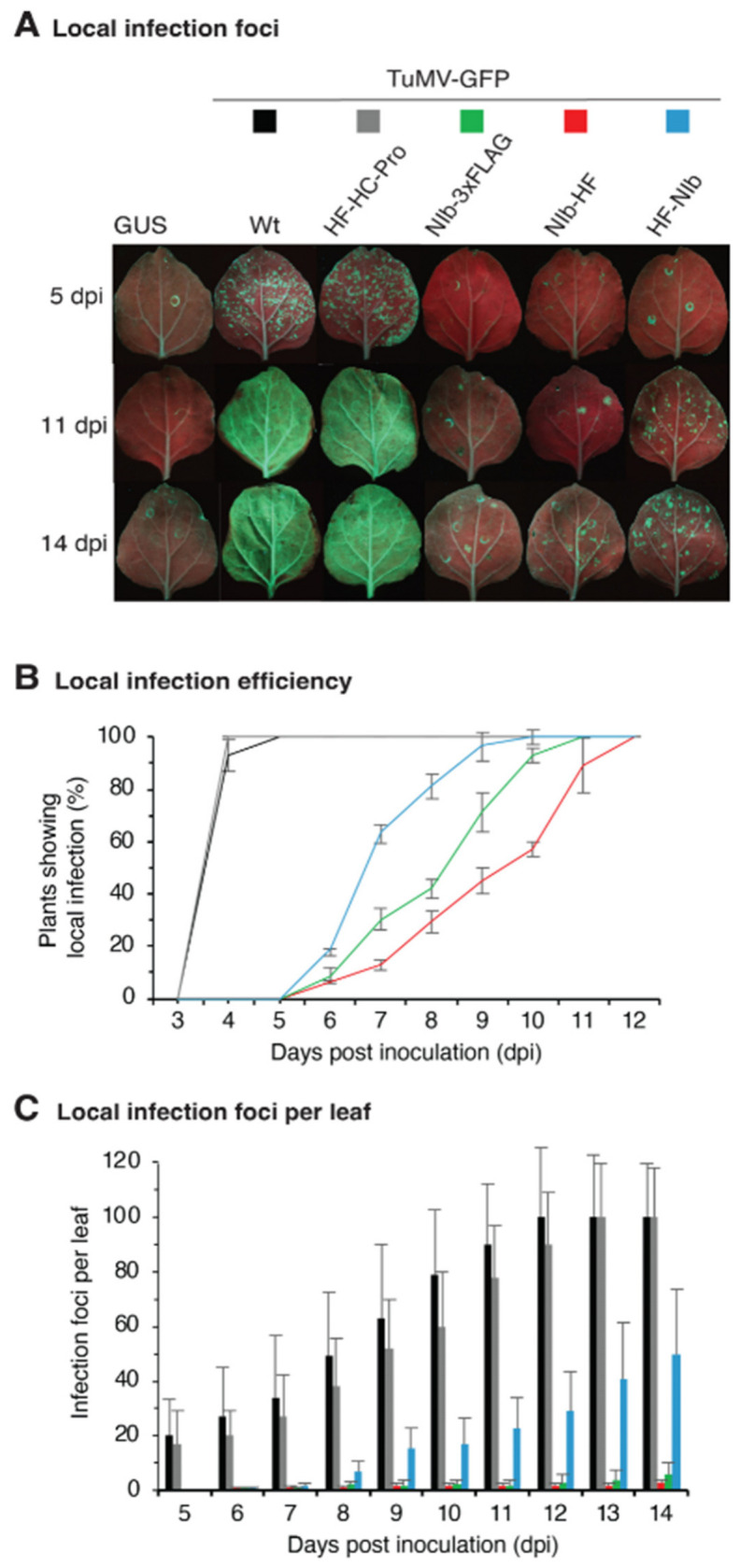
Local infection of *N. benthamiana* agroinfiltrated with TuMV-GFP clones (color-coded). The OD_A600_ at infiltration was 0.001. The experiment was repeated three times with 18 plants per treatment each time. (**A**) Pictures were taken at 5 dpi, 11 dpi, and 14 dpi under UV light. (**B**) Proportion of plants showing local infection from 3 to 12 dpi. (**C**) Number of local infection foci per leaf in days 5 to 14. In (**B**,**C**), measurements represent the average and standard error of three independent experiments and the colors correspond to the clones in (**A**).

**Figure 3:**
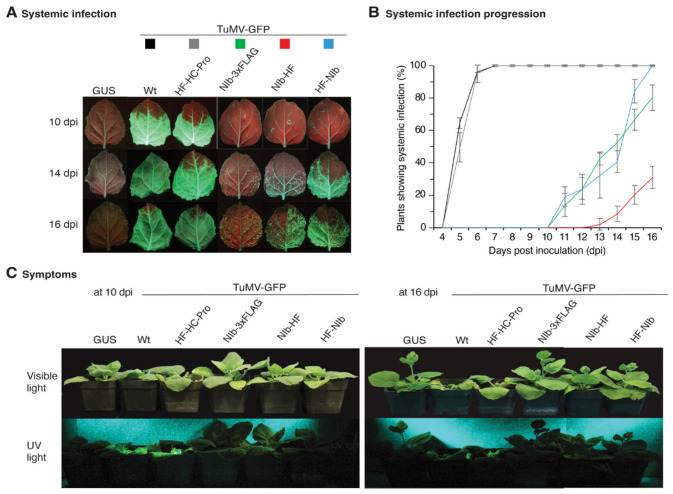
Systemic infection of *N. benthamiana* plants inoculated with TuMV-GFP derivatives. Local infection corresponding to these plants is in Figure 2. (**A**) Systemically infected leaves at 10, 14, and 16 dpi. TuMV-GFP derivatives are color-coded. (**B**) Proportion of plants showing systemic infection. Colors correspond to TuMV-GFP derivatives in (**A**). (**C**) Representative symptoms of plants under visible light and UV light at 10 dpi.

To assess their impact, different tags were added to HC-Pro and NIb genes separately within the infectious TuMV-GFP clone (Figure 1). Versions of TuMV-GFP with tagged HC-Pro or NIb were tested in *N. benthamiana* by agroinfiltration. TuMV-GFP and GUS were used as positive and negative controls, respectively. In this assay, local infection foci were visible by GFP at 4 days post-inoculation (dpi) in plants inoculated with TuMV-GFP (Figure 2A). The number of plants exhibiting local infection were recorded from 3 to 12 dpi, and the percentage of infected plants determined (Figure 2B). At 5 dpi and later, the number of local infection foci observed in leaves inoculated with the clone expressing HC-Pro tagged with 6xHis-3xFlag-HC-Pro at the N-terminal part (TuMV-GFP-HF-HC-Pro) were similar (17 ± 7 per leaf) to the number of infection foci observed in leaves inoculated with TuMV-GFP (20 ± 7 per leaf) (Figure 2C). Systemic infection was monitored by tracking the progression of GFP towards upper leaves. Plants with systemically infected leaves were counted for all the constructs from 4 to 16 dpi (Figure 3A). For TuMV-GFP, systemic infection was first detected at 5 dpi (Figure 3B) and systemic infection reached 100% at 7 dpi. A similar pattern of systemic infection was observed in plants inoculated with the clone expressing HC-Pro tagged with 6xHis-3xFlag-HC-Pro at the N-terminus (Figure 3B). Results were consistent across three independent experiments and indicate that tagging HC-Pro at the N-terminus did not affect local infection efficiency nor the establishment of systemic infection.

### 3.2. Tagging NIb Reduced and Delayed Local and Systemic Infection

In initial experiments, *N. benthamiana* plants were incubated at a 24 °C (day, 16 h) and 22 °C (night, 8 h) cycle after agroinfiltration. Local and systemic infections were readily detected in plants inoculated with TuMV or with the clone expressing HC-Pro tagged with 6xHis-3xFlag-HC-Pro. In contrast, clones in which NIb was tagged failed to establish local and systemic infection. Interestingly, local and systemic infections were observed when plants were incubated at a 22 °C (day, 16 h) and 18 °C (night, 8 h) cycle after agroinfiltration. These results showed that infection by clones expressing a tagged NIb is temperature-sensitive.

At the permissive temperature, for the clone expressing NIb tagged at the N-terminus with 6xHis-3xFLAG (TuMV-GFP-6xHis-3xFLAG-NIb), establishment of local infection was delayed by 6 days with respect to TuMV-GFP (Figure 2). Local infection foci were only detectable after 6 dpi (Figure 2A) and only in 20% of infiltrated plants (Figure 2B), and with an average of approximately two local foci per leaf (Figure 2C). That is a 90% reduction in local infection efficiency compared to TuMV-GFP. Additionally, it took 10 dpi for all inoculated plants to show at least one local infection foci. That was 6 days longer than the observed for TuMV-GFP (Figure 2B). A similar effect was detected for the establishment of systemic infection (Figure 3). For the clone expressing NIb tagged at the N-terminus with 6xHis-3xFLAG (TuMV-GFP-6xHis-3xFLAG-NIb), establishment of systemic infection was delayed compared to that typically observed for TuMV-GFP (Figure 3A). Systemic infection was first detected at 11 dpi in 20% of the inoculated plants. The proportion of systemically infected plants gradually increased, reaching 100% by 16 dpi, which was 9 days later than that observed for TuMV-GFP (Figure 3B). Furthermore, at 16 dpi, GFP indicating systemic infection covered only 10% of the leaves, compared to 95% observed for TuMV-GFP (Figure 3A). With respect to symptoms, plants inoculated with TuMV-GFP were stunted with respect to the negative control (GUS). In contrast, plants inoculated with tagged NIb showed no visible symptoms and grew similarly to that of the negative control, and no systemic GFP was detected in pictures taken under UV light (Figure 3C).

Similar results were obtained for clones expressing NIb tagged at the C-terminus with 6xHis-3xFLAG (TuMV-GFP-NIb-6xHis-3xFLAG) or with 3xFLAG (TuMV-GFP-NIb-3xFLAG) (Figure 3). Collectively, these results showed that tagging NIb significantly reduced local infection efficiency and delayed the establishment of both local and systemic infection in *N. benthamiana* (Figure 2 and Figure 3).

### 3.3. Tags on HC-Pro and NIb Are Genetically Stable

**Figure 4:**
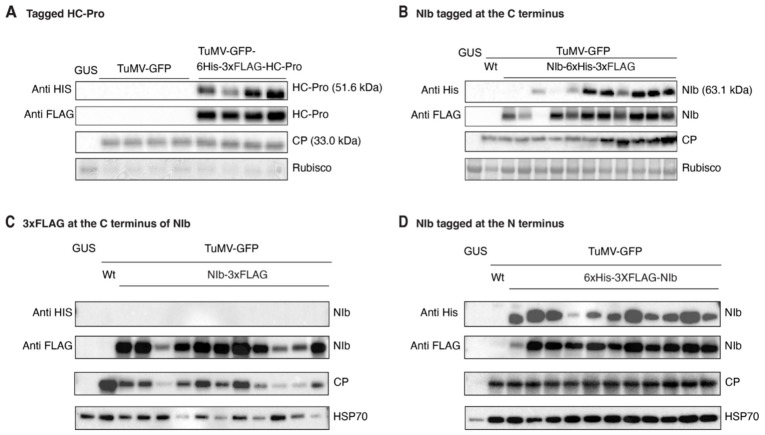
Protein accumulation in systemically infected leaves at 14 dpi. Anti-His and anti-FLAG antibodies were used to detect the His and FLAG tags fused to HC-Pro or NIb. Coat protein (CP) indicates TuMV accumulation. HSP70 (70 kDa) was used as a loading control. Rubisco was detected with Ponceau S staining and used as a second loading control. Panels from (**A**–**D**) indicate protein accumulation for each clone compared to TuMV-GFP and to plants infiltrated with a vector carrying GUS.

The epitope tags fused to HC-Pro and NIb could be lost during viral replication [51]. To determine the genetic stability of the epitope tags added to HC-Pro or NIb, we used western blotting to detect the HIS and the FLAG parts of the tag using anti-His and anti-FLAG antibodies [12] for the samples collected at 14 dpi from young non-inoculated leaves at the top of the plants. Antibodies to the coat protein (CP) [6] were used to confirm the presence of the TuMV, while anti-HSP70 and/or Rubisco were used as a loading control [19]. HC-Pro was readily detected with anti-His and anti-FLAG antibodies in all tested samples, and the viral CP accumulated to levels similar to those observed for TuMV-GFP (Figure 4A). When NIb was tagged at the C-terminal part with 6xHis-3xFLAG, NIb was readily detected with both anti-His and anti-FLAG antibodies. Only one of the samples lost the His, and only one of the samples lost the FLAG tag (Figure 4B). Similarly, NIb was readily detected when the tag was added at the N-terminal part, and the tag was not lost on any of the samples analyzed. (Figure 4C,D). Together, these results show that the tags added to HC-Pro and NIb at the N- or C-terminal part were maintained during the establishment of local and systemic infection.

### 3.4. Infection Efficiency Defects Are Independent from Silencing Suppression

**Figure 5:**
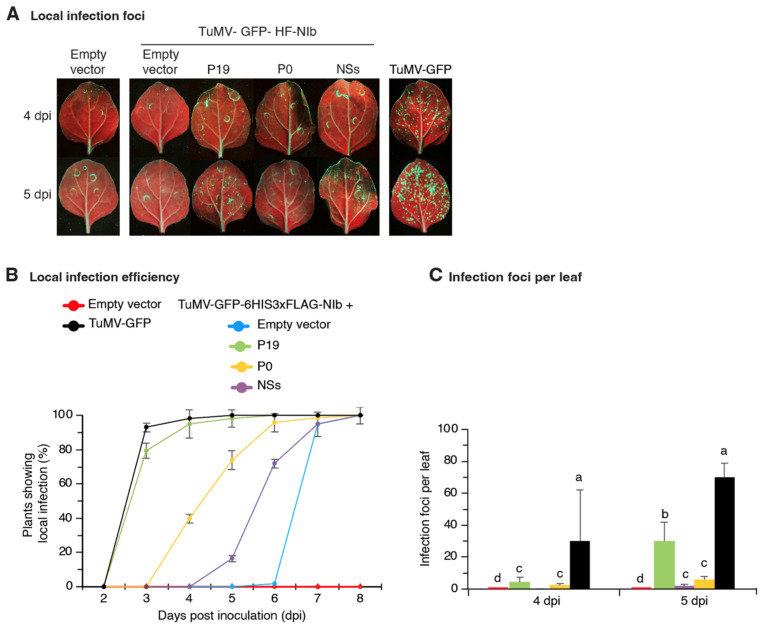
Heterologous silencing suppressor did not rescue the infectivity of TuMV-GFP expressing tagged NIb at the N-terminus (TuMV-HF-NIb). TuMV-GFP was used as a positive control. An empty vector (pMDC32) was used as a negative control. Silencing suppressors P19, P0, and NSs were provided through pMDC32 at an OD_A600_ = 0.25. For TuMV-GFP clones, the OD_A600_ at infiltration was 0.001. The experiment was repeated three times with 18 leaves infiltrated per treatment at each repetition. (**A**) Representative leaves showing local infection of *N. benthamiana* at 4 and 5 dpi. Pictures were taken under UV light. (**B**) Average percentage of plants exhibiting local infection. Suppressors are color-coded. (**C**) Average number of local infection foci per leaf. Suppressors are color-coded as in (**B**). Bars with the same letter are not statistically different (*p* = 0.01).

In *Potyvirus yituberosi* (potato virus Y), NIb has silencing suppression activity [52]. Thus, delays in virus replication described above could result from reduced amounts of HC-Pro or NIb available to suppress antiviral gene silencing. Under this scenario, the addition of a heterologous from TSBV [6], P0 from PLRV [53], and NSs from TSWV [19] by co-in suppressor is predicted to restore infection efficiency to clones carrying tagged NIb derivatives. To test this hypothesis, we provided silencing suppressors P19 filtration with TuMV-GFP carrying NIb tagged at the N-terminus with 6His-3xFLAG (TuMV-GFP-HF-NIb) (Figure 5). Infiltration with TuMV-GFP was used as a positive control. Infiltration with an empty vector construct only was used as a negative control. Local infection foci were detected and counted at 4 and 5 dpi (Figure 5A). Results showed the addition of P19, P0 or NSs did not accelerate local infection (Figure 5B) and was only 20% of that observed for TuMV-GFP (Figure 5C). With respect to co-infiltration with an empty vector, the presence of silencing suppressors, more notably P19, accelerated visibility of local infection (Figure 5B) and enhanced the number of local infection foci (Figure 5C). These results indicate that silencing suppressors enhanced the establishment of local infection by TuMV-GFP expressing NIb tagged at the N-terminus. However, the enhancement did not restore infection to TuMV-GFP levels. Thus, we conclude that defects in local and systemic infection observed (Figure 2 and Figure 3) in clones carrying NIb with a tag at the N- or C-terminus are not related to gene silencing.

### 3.5. Infection Efficiency in Arabidopsis thaliana

**Figure 6:**
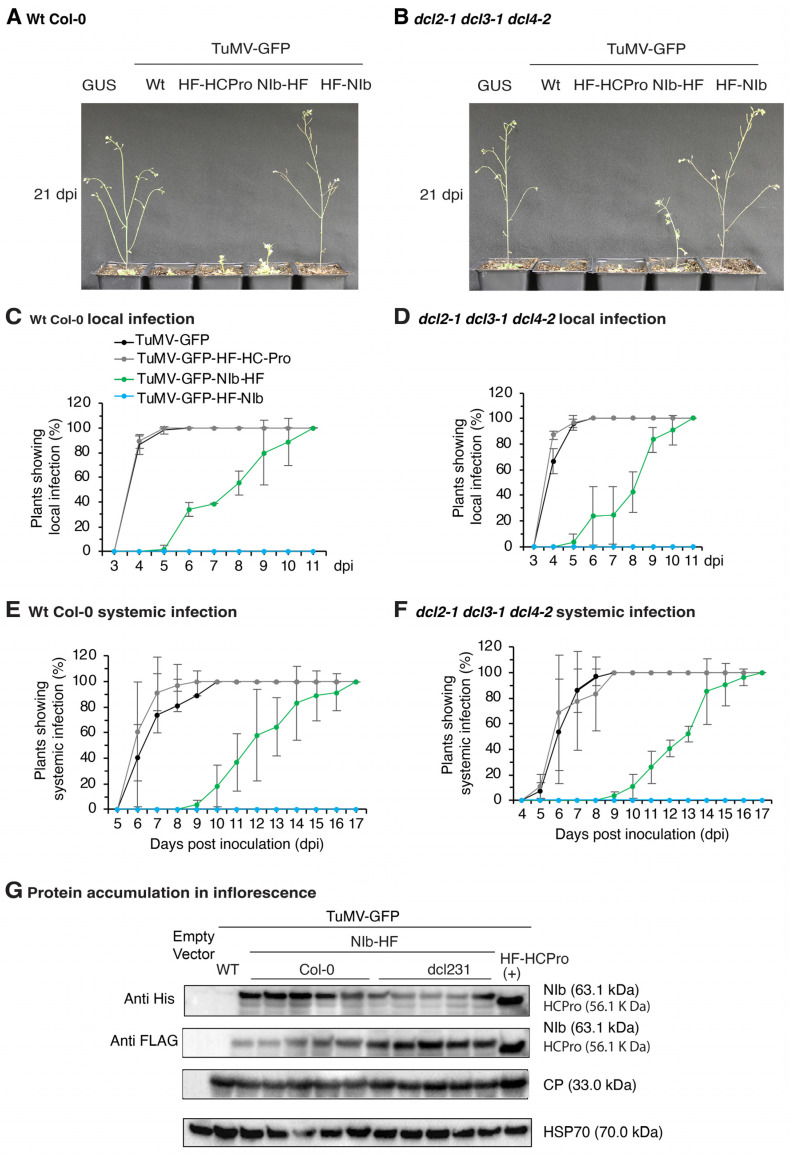
Pathogenicity, accumulation, and stability of TuMV-GFP derivatives carrying tagged HC-Pro (6XHis-3XFLAG at the N-terminus) or NIb at N- and C-terminus. Arabidopsis thaliana ecotype Col-0 wild-type and dcl2-1 dcl3-1 dcl4-2 triple mutant plants were inoculated by agroinfiltration (ODA600 = 0.1) of rosette leaves. The experiment was repeated three times with 18 plants per treatment each time. Values represent the average and standard error of the three repetitions. Vectors carrying GUS and TuMV-GFP were used as negative and positive controls. (**A**) Symptoms of wild-type (wt) plants and (**B**) dcl2-1 dcl3-1 dcl4-2 triple mutant plants at 21 dpi. Proportion (%) of plants showing local infection in (**C**) wild-type and (**D**) dcl2-1 dcl3-1 dcl4-2 triple mutant plants from 3 to 11 dpi. Clones and line graphs are color-coded. Proportion (%) of plants showing systemic infection of inflorescence in (**E**) wild-type (**F**) and dcl2-1 dcl3-1 dcl4-2 triple mutant plants from 5 to 17 dpi. (**G**) Viral coat protein (CP) and NIb accumulation in the inflorescence at 21 dpi as assessed by western blotting using anti-HIS and anti-FLAG antibodies. HSP70 was used as a loading control.

To determine whether the delayed infection phenotype is host-specific, clones with tagged NIb were tested in *Arabidopsis thaliana* ecotype Columbia-0 (Col-0). To further assess the role of antiviral gene silencing, the RNA silencing–deficient DICER-like triple mutant *dcl2-1 dcl3-1 dcl4-2* was used. TuMV-GFP and the derivative with HC-Pro tagged at the N-terminus were used as controls. Plants were inoculated by agroinfiltration of two rosette leaves. Both wild-type and *dcl2-1 dcl3-1 dcl4-2* triple mutant plants inoculated with TuMV-GFP or the derivative with HC-Pro tagged at the N-terminus showed 100% local infection at 5 dpi. Systemic infection initiated at 6 dpi and reached 100% by 9 dpi, with plants showing severe symptoms (Figure 6A–D). In contrast, wild-type and *dcl2-1 dlc3-1 dcl4-2* triple mutant plants inoculated with TuMV-GFP expressing tagged NIb at the N-terminus did not show visible symptoms, and signs of GFP indicating local or systemic infection were not detected up to 21 dpi (Figure 6A,B). By comparison, plants inoculated with TuMV-GFP expressing NIb tagged at the C-terminus exhibited delayed infection relative to TuMV-GFP (Figure 6C–F). Local infection was first observed at 6 dpi and reached 100% by 11 dpi, while systemic infection reached 100% at 17 dpi. Overall, plants showed mild symptoms at 21 dpi (Figure 6A,B).

In this assay, there was no difference in the pattern of infection or pathogenicity in wild-type versus dcl2-1 dcl3-1 dcl4-2 triple mutant plants (Figure 6A–D). Since removing the major antiviral DICER-like proteins did not rescue the virus pathogenicity, we conclude that reduced or delayed infection of TuMV-GFP derivatives with tagged NIb is not caused by the plant’s antiviral gene silencing response.

To further assess the genetic stability of the tags, systemically infected leaves were collected at 16 dpi for TuMV-GFP and TuMV-HF-HC-Pro, and at 20 dpi for TuMV-GFP-NIb-HF (Figure 6G). Western blot analysis using anti-His and anti-FLAG antibodies confirmed that both HC-Pro and NIb retained both the 6His part and the FLAG part of the tags, indicating that in plants showing delayed infection, it was not due to loss of the tags and reversion to wild-type NIb.

### 3.6. Structural Models of HC-Pro and NIb

**Figure 7:**
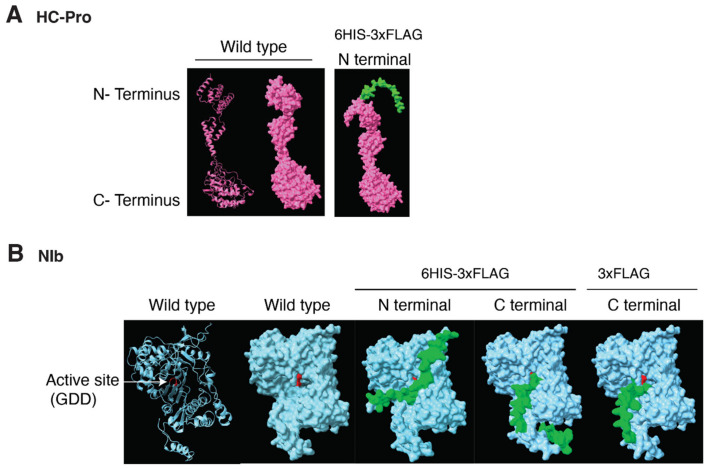
Secondary and three-dimensional structure predictions generated with AlphaFold2 for HC-Pro and NIb. (**A**) HC-Pro models with and without 6xHis-3xFlag (HF, marked in green) at the N-terminus. (**B**) NIb models. The RNA-dependent RNA polymerase active site (GDD) is highlighted in red. Epitope tags are shown in green, including 6xHis-3xFLAG at the N- and C-termini and a 3xFLAG tag at the C-terminus.

AlphaFold2 is a machine-learning-based tool that predicts protein structures from their amino acid sequences [38], and was used to generate 3D structural models of HC-Pro and NIb wild type and with tags added at the N- or C-terminal parts. Models revealed that the 6xHis-3xFLAG tag added to the N-terminus did not alter the 3D structure for HC-Pro (Figure 7A). The tag folds away and does not interfere with the overall protein folding or the active residues (Table 1) in the protein structure (Figure 7A). These observations are in agreement with the normal biological activity of the TuMV-GFP clone carrying a tagged HC-Pro (Figure 2, Figure 3 and Figure 6). In contrast, structures for NIb tagged with 6xHis-3xFLAG at the N- or C-terminus revealed a significant change in the structure and folded into the catalytic GDD motif, potentially impairing critical functions required during virus replication (Figure 7B and Table 2). These observations are in agreement with the temperature sensitivity and reduced pathogenicity of the TuMV-GFP clone carrying a tagged NIb (Figure 2, Figure 3 and Figure 6).

**Table 2:** Solvent-accessible surface area (SASA) analysis of the predicted structure of NIb with different tags at the N- and C-termini. Bold fonts indicate a significant (>1%) interference of the tag with the active site, nearby residues, or residues forming the geometric pore for substrate binding (pore-lining residues).

Protein and Tag	Percent Change in the Active Site	Percent Change in Residues Proximal to the Active Site	Percent Change in the Pore-Lining Region
HF-NIb	0	**−11.5**	**−11.7**
NIb-HF	−0.4	**−22.6**	**−12.1**
NIb-3xFlag	0	**−1.6**	**−8.2**
3xFlag-NIb	0	0	**−4.2**
NIb-Avi	0	0	**−3.8**
NIb-CaMBP	0	0	**−7.5**
NIb-MBP	0	**−8**	**−18.9**
NIb-SBP	**−6.9**	**−34.9**	**−31.5**
NIb-HRP	0	0	**−4.7**
SBP-NIb	0	0	−0.6
No effect at N-terminus	c-Myc, CBD, CLIP, GST, HA, His, S-tag, SNAP, V5, CaMBP, MBP, Avi, StrepII		
No effect at C-terminus	c-Myc, CBD, CLIP, GST, HA, His, S-tag, SNAP, V5, APEX2, BioID, BioID2, TurboID, Mini-TurboID		

Glutathione S-transferase protein (GST), Maltose Binding Protein (MBP), Calmodulin-binding peptide (CaMBP), Chitin-binding domain (CBD), Horseradish peroxidase (HRP).

### 3.7. Structural Models of NIb with Several Tags

**Figure 8:**
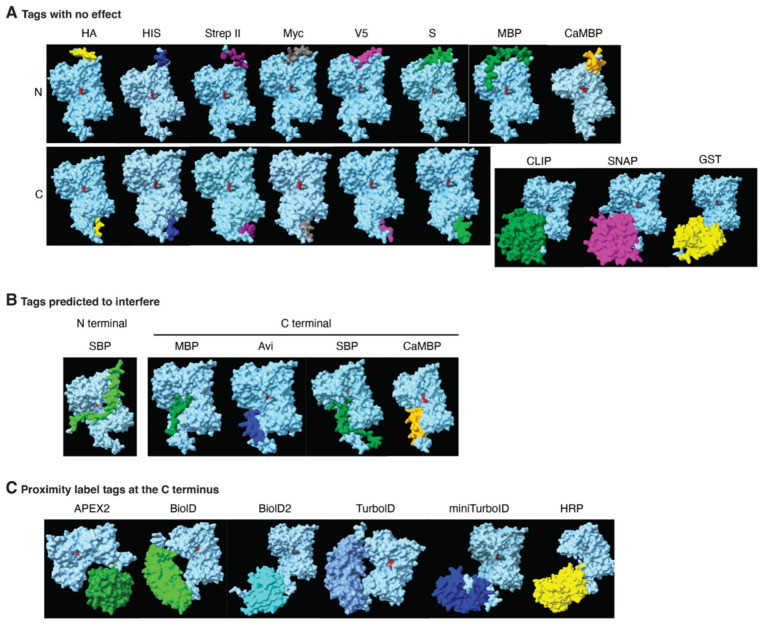
Predicted structures for NIb (is blue) with several tags (color coded). (**A**) Tags are at the N- or C-terminus and predicted not to affect NIb structure and function. (**B**) Tags predicted to alter the structure and function of NIb when added to the N- or C- terminus. (**C**) NIb models after the addition of proximity labeling tags at the C-terminus. All of them are predicted not to affect NIb structure and function.

The predicted structural models of HC-Pro and NIb are consistent with the pathogenicity properties observed for the corresponding TuMV-GFP derivatives carrying epitope tags, indicating that tag selection is critical for preserving native protein structure and function. Thus, we used AlphaFold2 to generate 3D structural models for NIb with several tags (Table S2) added to the N- or C-terminal parts. The potential for interference with the catalytic GDD motif was measured using solvent-accessible surface area (SASA) analysis that calculates the percentage of changes at the active site and residues proximal to the active site, and the pore-lining region. Results are indicated in Table 2 [40]. Models identified a group of tags predicted not to affect the overall NIb protein folding or function. These include HA, HIS, Strep II, Myc, V5, S, MBP, CaMBP at both C- and N-termini, as well as CLIP, SNAP, and GST when fused to the C-terminus (Figure 8A and Table 2). In contrast, tags predicted to interfere with NIb folding and function include SBP at both C- and N-termini, and MBP, Avi, and CaMBP at the C-terminus (Figure 8B and Table 2). The full representation of SASA analysis is represented in Table S3.

The use of proximity labeling tags is increasingly common [9], and we therefore modeled the effect of proximity labeling tags on NIb. AlphaFold2 structures indicated proximity labeling tags predicted not to interfere with protein folding or the overall structure of NIb. These include APEX2, BioID, BioID2, TurboID, and miniTurboID (Figure 8C and Table 2). HRP tag is predicted to interfere with NIb according to SASA analysis with 4.7 change in the pore-lining region (Figure 8C and Table 2).

### 3.8. Modeling the Effect of HIS and FLAG Tags on Other TuMV Proteins

**Figure 9:**
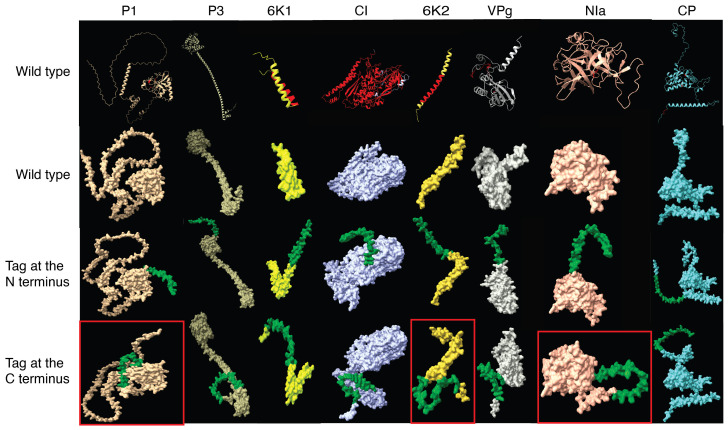
AlphaFold2 models for TuMV proteins in wild-type conditions and in the presence of a 6XHis-3XFLAG tag at the N- or C-terminus. Each protein is represented in a distinct color. The 6XHis–3XFlag tag is highlighted in green. When available, active site motifs are highlighted in red. The first row shows the predicted secondary structures of the wild-type proteins. The second row displays the corresponding three-dimensional structures of the wild-type proteins. The third and fourth rows show the predicted structures in the presence of the tag at the N- or C-terminus, respectively. The protein models that predict an effect on the protein structure when tagged with 6xHis-3xFLAG are marked with a red square.

HC-Pro and NIb structural models described above provide valuable information before initiating the sub-cloning process and biological characterization. We used AlphaFold2 to generate 3D structural models for all other TuMV proteins (P1, CI, VPg, 6K1, NIa, P3, and 6K2) when a 6xHis-3xFLAG tag is added to the N- or C-terminal parts (Figure 9). The amino acid sequences of the proteins are listed in Table S4. Criteria used to evaluate the impact of the tag included whether the tag overlapped with, blocked, or distorted the catalytic motif or regions critical for maintaining protein structure and function. Wild-type structures were compared to tagged versions, and the effect was predicted using solvent-accessible surface area (SASA) changes at the functional motifs and surrounding residues [40]. The known functions and functional motifs for TuMV proteins were determined from the published literature (Table 1) and highlighted in the secondary structure (Figure 9). No information was identified for the P3 active motif, and the whole sequence of the protein was used as a functional region. Structural predictions and SASA results showed significant differences for P1, 6K2, and NIa tagged at the C-terminus, with changes in the functional motif site of −26.8, −2.6, and −82.8, respectively. Differences in residues proximal to the active site (AS_5A) for these proteins were −8.8, 3.6, and 26.9, respectively (Table 3 and Figure 9). In contrast, no significant structural disruption was observed for P3, 6K1, CI, VPg, and CP at the C-terminal tagged part and for P1, P3, 6K1, 6K2, VPg, NIa-pro, and CP at the N-terminal tagged part (Table 3 and Figure 9). The SASA analysis is presented in Table S5.

## 4. Discussion

In the context of individual proteins or complete infectious clones, epitope tagging of viral proteins is a widely used approach for investigating virus–host interactions and for identification of host proteins interacting with viral proteins during infection [9,10]. It is commonly assumed that the incorporation of small tags has minimal impact on viral fitness [8,10]. Here, we constructed TuMV infectious clones with tags (6xHis-3xFLAG) on HC-Pro or NIb and tested their pathogenicity in model plants. Results identified limitations of protein tagging and provided a modeling strategy for predicting the effect of epitope tags on viral protein structure and function. Our results demonstrate that the effect of tagging is dependent on the viral protein and the way the tag folds with respect to the active site of the protein. For proteins expressed at low abundance, antibodies targeting a single tag are often inadequate for reliable detection. To improve sensitivity and to eliminate background during the purification process, tandem repeat arrays of widely used epitope tags, such as HA, MYC, Strep, His, and FLAG, can be used with the protein of interest [54,55]. Here, 6xHis combined with 3xFLAG was used to tag HC-Pro and NIb in TuMV. Fusing a 6xHis-3xFLAG tag at the N-terminus of HC-Pro did not affect pathogenicity nor the patterns of local and systemic infection. In a similar result, tagging *Potyvirus sacchari* (sugarcane mosaic virus, SCMV) HC-Pro with FLAG at the N-terminus had no effect on pathogenicity [8].

In contrast, when fusing the same tag to NIb either at the N- or C-terminus, or even only the 3xFLAG at the C-terminus, the infection became temperature-sensitive, and there was a delay in both local and systemic infection, both in *N. benthamiana* and *A. thaliana* at the permissive temperature (Figure 2 and Figure 3). TuMV derivatives expressing tagged NIb showed a substantial reduction in local infection efficiency, systemic spread, accumulated to lower levels, and caused mild symptoms compared to the observed for TuMV-GFP. In both cases, HC-Pro and NIb, the tags were maintained during the one cycle of infection needed to complete the experiment (Figure 4 and Figure 6G). These findings show contrasting effects of using the same tag on different proteins of the same virus (TuMV), and highlight the importance of careful selection of the epitope tags. These observations are in agreement with tagging tobacco etch virus VPg and NIa with twin Strep-tag at five different positions. In *N. benthamiana*, two of the five tagged clones lost infectivity, two induced milder symptoms, and only one induced symptoms similar to those observed for the wild-type virus [10].

To understand the results of pathogenicity assays and to improve selection of epitope tags, we implemented structural predictions using AlphaFold2 models and comparative analysis in the presence vs. the absence of tags. Changes in pathogenicity described correlated with 3D structural models generated using AlphaFold2. Models showed that HC-Pro fusing 6His-3xFLAG to the N-terminus of HC-Pro did not interfere with the active site motif (Figure 7A). This observation is in agreement with normal pathogenicity both in *N. benthamiana* and *A. thaliana* (Figure 2, Figure 3 and Figure 6). In sharp contrast, NIb adopts a more compact and structurally complex conformation, with multiple functional domains tightly organized around a centrally located active site containing the conserved GDD motif (Figure 7B). Structural models showed that fusing 6His-3xFLAG to the N- or C-terminal part of NIb altered the structure as the tag folded into the catalytic site (Figure 7B) and reduced the solvent-accessible surface area [40] (Table 2). This interference is consistent with the reduced pathogenicity in both *N. benthamiana* and *A. thaliana* (Figure 2, Figure 3 and Figure 6). In the normal course of infection, NIb is cleaved from the polyprotein by NIa cleavage both at the N- and C-termini (Figure 1) [1]. In our clones, the NIa cleavage site was maintained or restored after tagging NIb at the N- or C-terminus. However, we did not determine cleavage efficiency after adding the tag. Thus, there is no experimental evidence to rule out the possibility that reduced pathogenicity is due to reduced NIa cleavage efficiency after tagging NIb.

In positive-strand RNA viruses, the most structurally and functionally conserved protein is the RNA-dependent RNA polymerase (RdRp) responsible for virus replication, whose function depends on precise folding and on a highly conserved catalytic motif (GDD) [32]. Previous studies have shown that even minor insertions or structural perturbations within RdRp proteins can reduce viral replication efficiency or abolish infectivity completely [56]. Recent structural studies of plant viral RdRps have demonstrated that polymerase activation depends on precise conformational rearrangements triggered by viral RNA binding and that disruption of the structure can lock the polymerase in an inactive state [57]. This structural sensitivity provides an explanation for the effects observed on tagged NIb.

Previous studies showed that, out of the entire virus context, NIb as an individual protein tolerates tagging without significant functional disruption [58]. Our results showed that tagging NIb in the context of the entire virus interfered with activity. In this study, all NIb-tagged variants showed some degree of delayed replication and reduced pathogenicity both in *N. benthamiana* (Figure 2 and Figure 3) and in *A. thaliana* (Figure 6). One potential explanation is that the tagged NIb protein caused activation of host antiviral RNA defense, such as gene silencing. Natural examples of this phenomenon were described in arepaviruses, where spontaneous deletion of the HC-Pro silencing suppressor leads to attenuated, delayed, and low virus accumulation that can be rescued by trans-complementation with a strong silencing suppressor (P19) or in RNA silencing–deficient host backgrounds [59]. Similar observations were reported for engineered potyviruses lacking functional silencing suppressors, in which viral replication and movement remain possible but are strongly restricted by gene silencing [6,60]. Reduced pathogenicity of TuMV derivatives with tagged NIb was not rescued by heterologous silencing suppressors P19, P0, or NSs (Figure 5), nor in *dcl2-1 dcl2-1 dcl3-2* triple mutant plants defective in gene silencing (Figure 5). Both observations show that delayed infection and reduced pathogenicity when NIb was tagged are not related to antiviral gene silencing.

Based on results described above, we used structural models to predict the effect of the 6His-3xFLAG at the N- or C-terminal part of all other TuMV proteins, and the effect of several tags at the N- or C-terminal part of NIb. Results identified proteins predicted to be affected or not by 6xHis-3xFLAG tag (Figure 9), and tags predicted to affect, or not affect, NIb (Figure 8). These results provide valuable information before investing time and resources in tagging proteins of interest either individually or in the context of an infectious clone (Figure 1).

Together, our findings showed that protein modeling provides a rational and efficient alternative to traditional trial-and-error tagging strategies. This approach significantly reduces the risk of viral attenuation, accelerates infectious clone development, and improves the biological relevance of downstream interaction studies.

## 5. Conclusions

The TuMV-GFP infectious clones with epitope tags fused to individual proteins are essential to enable real-time visualization of viral movement, accurate subcellular localization, and co-immunoprecipitation of virus–host complexes directly from infected tissues and to reduce artifacts associated with transient expression systems. We developed TuMV derivatives, in which HC-Pro or NIb were tagged with 6xHis-3xFLAG, and tested their infectivity in model plants. Results showed contrasting effects. No changes in pathogenicity were observed in the clone with tagged HC-Pro, and no structural changes were detected in 3D models (Figure 7A). In contrast, temperature sensitivity, reduced infection efficiency, and reduced pathogenicity were observed in the clone with tagged NIb, and significant structural changes were detected in 3D models (Figure 7B). Structural modeling using AlphaFold2 and pathogenicity assays provides a rational framework for epitope tag selection in viruses.

## Figures and Tables

**Table 1:** Roles and active catalytic sites or functional motifs of potyvirus proteins.

Protein	Function	Key Active Motif/Residue	Size (aa)	Size (kDa)	Refs.
P1	Serine protease (self-cleavage)	Ser–His–Asp catalytic triad	362	42	[42]
HC-Pro	Cysteine protease, RNA silencing suppression	KITC, PTK, F(Y)RNK, IGN, CC/SC, YXVG/G	457	52	[26]
P3	Pathogenicity determinant, movement	No defined	355	48	
6K1	Membrane anchoring of replication complex	Hydrophobic transmembrane domain QSEQELERIIAFVALVLMMF	52	6	[43,44]
CI	Cylindrical inclusion helicase	Potyvirid-P3 superfamily (aa 2–57), DEAD-like helicases superfamily (73–230), helicase superfamily (246–368) and potyviridae polyprotein superfamily (386–644)	644	70	[45]
6K2	Form replication vesicles in the ER (membrane scaffold protein, not an enzyme)	Hydrophobic transmembrane domain (20–42)	53	6	[46]
VPg	Primer for replication	Tyr residue (RNA linkage), NTP-binding region	192	25	[47]
NIa-pro	Cysteine protease	His–Asp–Cys catalytic triad	243	37	[48,49]
NIb	Replication, avirulence factor	GDD	549	58	[31]
CP	Virion formation, cell-to-cell, systemic movement	DAG motif, R178 and D222	288	33	[50]

**Table 3:** Solvent-accessible surface area (SASA) analysis of the predicted structure of other potyviral proteins with 6xHis-3xFLAG tags at N- and C-termini. Bold fonts indicate a notable (>1%) predicted tag interference with the protein structure. “-” indicates that the data are not available due to the lack of a well-defined substrate-binding pore. The entire SASA analysis is in Table S5.

Protein and Tag Position	Percent Change in the Active Site	Percent Change in Residues Proximal to the Active Site	Percent Change in the Pore-Lining Region
P1-C	**−26.8**	**−8.8**	-
P3-N	−0.3	−0.3	-
P3-C	−0.2	−0.2	-
6K1-N	−0.3	**−3.6**	-
6K1-C	0	**−1.3**	-
CI-N	−0.2	−0.2	-
CI-C	−0.2	−0.2	-
6K2-C	**−2.6**	**−3.6**	-
VPg-C	0	−0.1	-
NIa-C	**−82.8**	**−26.9**	**−18.5**
CP-N	0	**−3.8**	-
No effect	P1-N, 6K2-N, VPg-N, NIa-N, CP-C		

## Data Availability

The in-house Python 3.12.5, Bash 5.2, and R scripts used in the analyses are available upon request and as Supplemental Material S6 (run_sasa.r) and S7 (funs.R).

## Supplementary Materials

The following supporting information can be downloaded at https://www.mdpi.com/article/10.3390/pathogens15060611/s1, Table S1: Primers used to generate the constructed clones. Table S2: List of epitope tags. Table S3: Solvent-accessible surface area (SASA) analysis of the predicted structure of NIb with different tags at N- and C-terminus. Red fonts indicate an interference of the tag with the active site. Table S4: Sequences of TuMV proteins tagged with 6xHis-3xFlag at both C- and N-termini, the active catalytic sites or motifs are marked in red. Table S5: Solvent-accessible surface area (SASA) analysis of the predicted structure of other potyviral proteins with 6xHis-3xFLAG tags at N- and C-termini. Bold fonts indicate a notable (>1%) predicted tag interference with the protein structure. NA indicates the data are not available due to the lack of a well-defined substrate-binding pore. S6: run_sasa.r. S7: funs.R.
