# Supplementary material for: Contrasting Effects of Tagging Turnip Mosaic Virus Proteins
Source: Pathogens. 2026 Jun 8;15(6):611. doi: 10.3390/pathogens15060611 (PMC13305232; doi:10.3390/pathogens15060611)
Supplement: Supplementary file 1 [file pathogens-15-00611-s001.zip › Table_S1_Primers.pdf]

Table S1: Primers used to generate the constructed clones

Primer	Sequence (5′→3′)
111	AGGACGGTGCACAGAATATGC
674	GAAGTTGGCTCCTGCtgcCTTGTCATCGTCATCCTTGTAGTCGATGTCATGATCTTTATAATCACCGTCATGGTCTTTGTAGTCGTGATGGTGATGGTGATGaccTGCCTGGTGATAGACACAgctagcACTAAAGTGCAC
675	GTGCACTTTAGTgctagcTGTGTCTATCACCAGGCAggtCATCACCATCACCATCACGACTACAAAGACCATGACGGTGATTATAAAGATCATGACATCGACTACAAGGATGACGATGACAAGgcaGCAGGAGCCAACTTC
110	CGCCTGATTCTGTTGTGACAC
780	caccGCGATGATTGAGTCGTGGGG
719	ATCGCAAGACCGGCAACAGG
833	AGCCTCAGCACCATCTTCATAATCC
834	GACTACAAAGACCATGACGGTGATTATAAAGATCATGACATCGACTACAAGGATGACGATGACAAGtgtgtttatcaccaggcagg
835	CATCACCATCACCATCACGACTACAAAGACCATGACGGTGATTATAAAGATCATGACATCGACTACAAGGATGACGATGACAAGtgtgtttatcaccaggcagg
1093	accCATCACCATCACCATCACGACTACAAAGACCATGACGGTGATTATAAAGATCATGACATCGACTACAAGGATGACGATGACAAGcagcagaatcggtggatgttcgagc
1094	TTGTGCGTAGACTGCCGTGCTG
