# Supplementary material for: Contrasting Effects of Tagging Turnip Mosaic Virus Proteins
Source: Pathogens. 2026 Jun 8;15(6):611. doi: 10.3390/pathogens15060611 (PMC13305232; doi:10.3390/pathogens15060611)
Supplement: Supplementary file 1 [file pathogens-15-00611-s001.zip › Table_S2_Tags.pdf]

Table S2. List of epitope tags.

Tag name	Amino acid sequence	Length (aa)
Poly-Arg	RRRRR	5
HIS	HHHHHH	6
FLAG	DYKDDDDK	8
3x FLAG	DYKDHDGDYKDHDIDYKDDDDK	22
Strep II	WSHPQFEK	8
HA	YPYDVPDYA	9
c-myc	EQKLISEEDL	10
V5	GKPIPNPLLGLDST	14
MBP	MASMTGGQQMGRDLYDDDDKDPDYE	25
Avi	GLNDIFEAQKIEWHE	16
S-	KETAAAKFERQHMDS	14
SBP	MDEKTTGWRGGHVVEGLAGELEQLRARLEHHPQGQREP	37
CBD	TNPGVSAWQVNTAYTAGQLVTYNGKTYKCLQPHTSLAGWEPS NVPALWQLQ	50
CaMBP	KRRWKKNFIAVSAANRFKKISSSGAL	27
CLIP-tag	MDKDCEMKRTTLDSPLGKLELSGCEQGLHRIIFLGKGTSAADAV EVPAPAAVLGGPEPLIQATAWLNAYFHQPEAIEEFPVPALHHPV FQQESFTRQVLWKLLKVVKFGEVISESHLAALVGNPAATAAVNT ALDGNPVPILIPCHRVVQGDSDVGPYLGGLAVKEWLLAHEGHR LGKPGLG	159

Large fusion tags

Tag	Amino acid sequence	Length (aa)
GST	MSPILGYWKIKGLVQPTRLLLEYLEEKYEEHLYERDEGDKWRNKKFELGLEF PNLPYYIDGDVKLTQSMAIIRYIADKHNMLGGCPKERAEISMLEGAVLDIRY GVSRIAYSKDFETLKVDFLSKLPEMLKMFEDRLCHKTYLNGDHVTHPDFML YDALDVVLYMDPMCLDAFPKLVCFKKRIEAIPQIDKYLKSSKYIAWPLQGW QATFGGGDHPPKSD	211
SNAP	MDKDCEMKRTTLDSPLGKLELSGCEQGLHEIKLLGKGTSAADAVEVPAPAA VLGGPEPLMQATAWLNAYFHQPEAIEEFPVPALHHPVFQQESFTRQVLWKL LKVVKFGEVISYQQLAALAGNPAATAAVKTALSGNPVPILIPCHRVVSSSGA VGGYEGGLAVKEWLLAHEGHRLGKPGLG	182

Proximity labeling tags

Tag	Amino acid sequence	Plasmid
APEX2	GKSYPTVSADYQDAVEKAKKKLRGFIAEKRCAPLMLR LAFHSAGTFDKGTKTGGPFGTIKHPAELAHSANNGLDI AVRLLEPLKAEFPILSYADFYQLAGVVAVEVTGGPKVP FHPGREDKPEPPPEGRLPDPTKGSDHLRDVFGKAMGL TDQDIVALSGGHTIGAAHKERSGFEGPWTSNPLIFDNS YFTELLSGEKEGLLQLPSDKALLSDPVFRPLVDKYAADE DAFFADYAEAHQKLSELGFADA	Addgene #79057

HRP	QLTPTFYDNSCPNVSNIVRDTIVNELRSDPRIAASILRLH FHDCFVNGCDASILLDNTTSFRTEKDAFGNANSARGFP VIDRMKAAVESACPRTVSCADLLTIAAQQSVTLAGGPS WRVPLGRRDSLQAFLDLANANLPAPFFTLPQLKDSFR NVGLNRSSDLVALSGGHTFGKNQCRFIMDRLYNFSNT GLPDPTLNTTYLQTLRGLCPLNGNLSALVDFDLRTPTIF DNKYYVNLEEQKGLIQSDQELFSSPNATDTIPLVRSFAN STQTFFNAFVEAMDRMGNITPLTGTQGQIRLNCRVVN SNS	Addgene #73152
BioID	KDNTVPLKLIALLANGEFHSGEQLGETLGMSRAAINK HIQTLRDWGVDVFTVPGKGYSLPEPIQLLNAKQILGQL DGGSVAVLPVIDSTNQYLLDRIGELKSGDACIAEYQQA GRGGRGRKWFSPFGANLYLSMFWRLEQGPAAAIGLSL VIGIVMAEVLRKLGADKVRVKWPNDLYLQDRKLAGIL VELTGKTGDAAQIVIGAGINMAMRRVEESVVNQGWIT LQEAGINLDRNTLAAMLIRELRAALELFEQEGLAPYLS RWEKLDNFINRPVKLIIGDKEIFGISRGIDKQGALLLEQ DGIIKPWMGGEISLRSAEK	Addgene #35700
BioID2	FKNLIWLKEVDSTQERLKEWNVSYGTALVADRQTKGR GGLGRKWLSQEGGLYFSFLLNPKEFENLLQLPLVLGLS VSEALEEITEIPFSLKWPNDVYFQEKKVSGVLCELSKDK LIVGIGINVNQREIPEEIKDRATTLYEITGKDWDRKEVLL KVLKRISENLKKFKEKSFKEFKGKIESKMLYLGEEVKLL GEGKITGKLVGLSEKGGALILTEEGIKEILSGEFSLRRS	Addgene #74224
TurboID	MKDNTVPLKLIALLANGEFHSGEQLGETLGMSRAAIN KHIQTLRDWGVDVFTVPGKGYSLPEPIPLLNAKQILGQ LDGGSVAVLPVVDSTNQYLLDRIGELKSGDACIAEYQQ AGRGSRGRKWFSPFGANLYLSMFWRLKRGPAAIGLGP VIGIVMAEALRKLGADKVRVKWPNDLYLQDRKLAGIL VELAGITGDAAQIVIGAGINVAMRRVEESVVNQGWITL QEAGINLDRNTLAATLIRELRAALELFEQEGLAPYLPR WEKLDNFINRPVKLIIGDKEIFGISRGIDKQGALLLEQD GVIKPWMGGEISLRSAE	Addgene #107177
Mini TurboID	IPLLNAKQILGQLDGGSVAVLPVVDSTNQYLLDRIGEL KSGDACIAEYQQAGRGSRGRKWFSPFGANLYLSMFWR LKRGPAAIGLGPVIGIVMAEALRKLGADKVRVKWPND LYLQDRKLAGILVELAGITGDAAQIVIGAGINVAMRRV EESVVNQGWITLQEAGINLDRNTLAAMLIRELRAALEL FEQEGLAPYLSRWEKLDNFINRPVKLIIGDKEIFGISRGI DKQGALLLEQDGVIKPWMGGEISLRSAEK	Addgene# 107178

GST glutathione S-transferase protein, MBP Maltose Binding Protein, CaMBP Calmodulin-binding peptide, Chitin-binding domain (CBD)
