# Supplementary material for: Contrasting Effects of Tagging Turnip Mosaic Virus Proteins
Source: Pathogens. 2026 Jun 8;15(6):611. doi: 10.3390/pathogens15060611 (PMC13305232; doi:10.3390/pathogens15060611)
Supplement: Supplementary file 1 [file pathogens-15-00611-s001.zip › Table_S3_SASA_NIb_1.pdf]

Table S3. Solvent-accessible surface area (SASA) analysis of the predicted structure of NIb with different tags at N- and C-terminus. Red fonts indicate an interference of the tag with the active site.

	Active.site[a]	Wotail.AS[b]	AS_5A[c]	Wotail.AS5A[d]	Pore.lining[e]	Wotail.pore[f]	%diff.AS[g]	%diff.AS_5A[h]	%diff.pore.lining[i]
3xFlag-NIb	95.1	95.1	352	352	1483.6	1549.4	0	0	-4.2
HF-NIb	99.9	99.9	306	345.6	1390.8	1575	0	-11.5	-11.7
NIb-HF	88.5	88.9	220.4	284.6	1362.5	1550.7	-0.4	-22.6	-12.1
NIb-3xFlag	100.5	100.5	357.6	363.5	1411.6	1537.9	0	-1.6	-8.2
NIb-Avi	103.6	103.6	359.2	359.2	1516.5	1576.7	0	0	-3.8
NIb-CaMBP	102.3	102.3	358.9	358.9	1451.7	1568.6	0	0	-7.5
NIb-HRP	85.3	85.3	277	277	1490	1564.2	0	0	-4.7
NIb-MBP	102	102	330.4	359.1	1264	1558.7	0	-8	-18.9
NIb-SBP	94.2	101.1	229.1	351.6	1070.5	1563.6	-6.9	-34.9	-31.5
SBP-NIb	99.7	99.7	353.8	353.8	1531	1540.3	0	0	-0.6
c-myc-NIb	95.5	95.5	348.5	348.5	1582.7	1582.7	0	0	0
CaMBP-NIb	93.9	93.9	355.7	355.7	1551.1	1551.1	0	0	0
CBD-NIb	96.8	96.8	308.7	308.7	1565.1	1565.1	0	0	0
CLIP-NIb	92.3	92.3	290	290	1579.8	1579.8	0	0	0
GST-NIb	90.1	90.1	276.2	276.2	1556.9	1556.9	0	0	0
HA-NIb	100.2	100.2	356.2	356.2	1558	1558	0	0	0
His-NIb	89.4	89.4	284.4	284.4	1585.2	1585.2	0	0	0
MBP-NIb	98.7	98.7	349.3	349.3	1580.6	1580.6	0	0	0
Avi-NIb	99.3	99.3	330.5	330.5	1574.8	1574.8	0	0	0
NIb-APEX2	84.2	84.2	275.2	275.2	1596.7	1596.7	0	0	0
NIb-BioID	98.4	98.4	342.3	342.3	1583.9	1583.9	0	0	0
NIb-BioID2	89.3	89.3	286.6	286.6	1565.2	1565.2	0	0	0
NIb-c-myc	98.5	98.5	357	357	1582.5	1582.5	0	0	0

NIb-CBD	99.7	99.7	339	339	1557.1	1557.1	0	0	0
NIb-CLIP	76.2	76.2	310.1	310.1	1529.1	1529.1	0	0	0
NIb-GST	101.9	101.9	353.3	353.3	1572.6	1572.6	0	0	0
NIb-HA	99.3	99.3	353.9	353.9	1571.3	1571.3	0	0	0
NIb-His	96.1	96.1	308.2	308.2	1593.7	1593.7	0	0	0
NIb-Mini-TurboID	98.1	98.1	318.3	318.3	1583.3	1583.3	0	0	0
Nib-S-Tag	100.8	100.8	353.7	353.7	1571.4	1571.4	0	0	0
NIb-SNAP	88.4	88.4	273.4	273.4	1571.4	1571.4	0	0	0
NIb-V5	99.2	99.2	344	344	1587.8	1587.8	0	0	0
S-tag-NIb	91.8	91.8	288.5	288.5	1586.9	1586.9	0	0	0
Snap-Tag-NIb	86	86	282.5	282.5	1582.9	1582.9	0	0	0
StrepII-NIb	101.2	101.2	358.9	358.9	1540.9	1540.9	0	0	0
v5-NIb	90.3	90.3	287	287	1582.4	1582.4	0	0	0
NIb-TurboID	95.8	95.8	329.9	329.9	1580.5	1580.5	0	0	0

[a] Values for residues that form the active site.
[b] Without tail Active.site.
[c] Residues proximal to the active site without tail.
[d] Residues proximal to the active site.
[e] Values for residues that line the pore.
[f] Values for residues that line the pore without tail.
[g] Percent change in the active site.
[h] Percent change in residues proximal to the active site.
[i] Percent change in the pore-lining region.
