# Supplementary material for: Contrasting Effects of Tagging Turnip Mosaic Virus Proteins
Source: Pathogens. 2026 Jun 8;15(6):611. doi: 10.3390/pathogens15060611 (PMC13305232; doi:10.3390/pathogens15060611)
Supplement: Supplementary file 1 [file pathogens-15-00611-s001.zip › Table_S4_otherPotyvirusproteins.pdf]

Table S4. Sequences of TuMV proteins tagged with 6xHis-3xFlag at both C- and N-termini, the active catalytic sites or motifs are marked in red.

Protein	Amino acid sequence	N- terminal tagging	C-Terminal tagging
P1	MAAVTFASAITNAITSKPALTGMVQFGS<br>FPPMPLRSTTVTTVATSVAQPKLYTVQF<br>GSLDPVVVKSGAGSLAKATRQQPNVEI<br>DVSLSEAAALEVAKPRSNAVLRMHEEA<br>NKERALFLDWEASLKRSSYGIAEDEKV<br>VMTTHGVSKIVPRSSRAMKLKRARERR<br>RAQQPIILKWEPKLSGISIGGGLSASVIEA<br>EEVRTKWPLHKTPSMKKRTVHRICKMN<br>DQGVDMLTRSLVKIFKTKSANIEYGKKS<br>IKVDFIRKERTKFARIQVAHLLGKRAQR<br>DLLTGMEENHFIDILSKYSGNKTTINPG<br>VVCAGWSGIVVGNGILTQKRSRSPSEAF<br>VIRGEHEGKLYDARIKVTRTMSHKIVHF<br>S	HHHHHHDYKDHDGDYKDHDIDYKDDDDK<br>MAAVTFASAITNAITSKPALTGMVQFGSFPP<br>MPLRSTTVTTVATSVAQPKLYTVQFGSLDP<br>VVVKSGAGSLAKATRQQPNVEIDVSLSEAA<br>ALEVAKPRSNAVLRMHEEANKERALFLDW<br>EASLKRSSYGIAEDEKVVMTTHGVSKIVPRS<br>SRAMKLKRARERRRAQQPIILKWEPKLSGIS<br>IGGGLSASVIEAEEVRTKWPLHKTPSMKKR<br>TVHRICKMNDQGVDMLTRSLVKIFKTKSAN<br>IEYGKKSIKVDFIRKERTKFARIQVAHLLGK<br>RAQRDLLTGMEENHFIDILSKYSGNKTTINP<br>GVVCAGWSGIVVGNGILTQKRSRSPSEAFVI<br>RGEHEGKLYDARIKVTRTMSHKIVHFS	MAAVTFASAITNAITSKPALTGMVQFGSFPP<br>MPLRSTTVTTVATSVAQPKLYTVQFGSLDP<br>VVVKSGAGSLAKATRQQPNVEIDVSLSEAA<br>ALEVAKPRSNAVLRMHEEANKERALFLDW<br>EASLKRSSYGIAEDEKVVMTTHGVSKIVPRS<br>SRAMKLKRARERRRAQQPIILKWEPKLSGIS<br>IGGGLSASVIEAEEVRTKWPLHKTPSMKKR<br>TVHRICKMNDQGVDMLTRSLVKIFKTKSAN<br>IEYGKKSIKVDFIRKERTKFARIQVAHLLGK<br>RAQRDLLTGMEENHFIDILSKYSGNKTTINP<br>GVVCAGWSGIVVGNGILTQKRSRSPSEAFVI<br>RGEHEGKLYDARIKVTRTMSHKHHHHHHD<br>YKDHDGDYKDHDIDYKDDDDKIVHFS
HC-Pro	AAGANFWKGFDRCFLAYRSDNREHTC<br>YSGLDVTECGEVAALMCLAMFPCGKIT<br>CPDCVTDSELSQGQASGPSMKHRLTQL<br>RDVIKSSYPRFKHAVQILDRYEQSLSSA<br>NENYQDFAEIQSISDGVEKAAFPHVNKL<br>NAILIKGATVTGEEFSQATKHLLEIARYL<br>KNRTENIEKGSLKSFRNKISQKAHINPTL<br>MCDNQLDRNGNFIWGERGYHAKRFFS<br>NYFEIIDPKKGYTQYETRAVPNGSRKLA<br>IGKLIVPTNFEVLREQMKGEPVEPYPVT<br>VECVSKLQGDFVHACCCVTTESGDPVL<br>SEIKMPTKHHLVIGNSGDPKYIDLPEIEE<br>NKMYIAKEGYCYINIFLAMLVNVKESQ<br>AKEFTKVVRDKLVGELGKWPTLLDVAT<br>ACYFLKVFYPDVANAELPRMLVDHKTK<br>IIHVVDSYGSLSTGYHVLKTNTVEQLIKF<br>TRCNLESSLKHYRVG	HHHHHHDYKDHDGDYKDHDIDYKDDDDK<br>AAGANFWKGFDRCFLAYRSDNREHTCYSG<br>LDVTECGEVAALMCLAMFPCGKITCPDCVT<br>DSELSQGQASGPSMKHRLTQLRDVIKSSYP<br>RFKHAVQILDRYEQSLSSANENYQDFAEIQS<br>ISDGVEKAAFPHVNKLNAILIKGATVTGEEF<br>SQATKHLLEIARYLKNRTENIEKGSLKSFRN<br>KISQKAHINPTLMCDNQLDRNGNFIWGERG<br>YHAKRFFSNYFEIIDPKKGYTQYETRAVPN<br>GSRKLAIGKLIVPTNFEVLREQMKGEPVEPY<br>PVTVECVSKLQGDFVHACCCVTTESGDPVL<br>SEIKMPTKHHLVIGNSGDPKYIDLPEIEENK<br>MYIAKEGYCYINIFLAMLVNVKESQAKEFT<br>KVVRDKLVGELGKWPTLLDVATACYFLKV<br>FYPDVANAELPRMLVDHKTKIIHVVDSYGS<br>LSTGYHVLKTNTVEQLIKFTRCNLESSLKHY<br>RVG	AAGANFWKGFDRCFLAYRSDNREHTCYSG<br>LDVTECGEVAALMCLAMFPCGKITCPDCVT<br>DSELSQGQASGPSMKHRLTQLRDVIKSSYP<br>RFKHAVQILDRYEQSLSSANENYQDFAEIQS<br>ISDGVEKAAFPHVNKLNAILIKGATVTGEEF<br>SQATKHLLEIARYLKNRTENIEKGSLKSFRN<br>KISQKAHINPTLMCDNQLDRNGNFIWGERG<br>YHAKRFFSNYFEIIDPKKGYTQYETRAVPN<br>GSRKLAIGKLIVPTNFEVLREQMKGEPVEPY<br>PVTVECVSKLQGDFVHACCCVTTESGDPVL<br>SEIKMPTKHHLVIGNSGDPKYIDLPEIEENK<br>MYIAKEGYCYINIFLAMLVNVKESQAKEFT<br>KVVRDKLVGELGKWPTLLDVATACYFLKV<br>FYPDVANAELPRMLVDHKTKIIHVVDSYGS<br>LSTGYHVLKTNTVEQLIKFTRCNLESSLKHH<br>HHHHHDYKDHDGDYKDHDIDYKDDDDKY<br>RVG
P3	GTEWEDTHGSSNIDNPQWCIKRLIKGVY<br>KPKQLKEDMLANPFLPLYALLSPGVILA<br>FYNSGSLEYLMNHYIRVDSNVAVLLVV<br>LKSLAKKVSTSQSVLAQLQIIERSLPELIE<br>AKANVNGPDDAATRACNRFMGMLLH<br>MAEPNWELADGGYTILRDHSISILEKSY<br>LQILDEAWNELSWSERCAIRYYSSKQAI<br>FTQKDLPMKSEADLGGRYSVSVMSSYE<br>RSKQCMKSVHSSIGNRLRSSMSWTSSK<br>VSNSVCRTINYLVPDVFKFMNVLVCISL<br>LIKMTAEANHIVTTQRRLKLDVEETERR<br>KIEWELAFHHAILTQSAGQHPTIDEFRA<br>YIADKAPHLSEHIEPEEKAVVHQ	HHHHHHDYKDHDGDYKDHDIDYKDDDDK<br>GTEWEDTHGSSNIDNPQWCIKRLIKGVYKP<br>KQLKEDMLANPFLPLYALLSPGVILAFYNS<br>GSLEYLMNHYIRVDSNVAVLLVVLKSLAK<br>KVSTSQSVLAQLQIIERSLPELIEAKANVNGP<br>DDAATRACNRFMGMLLHMAEPNWELADG<br>GYTILRDHSISILEKSYLQILDEAWNELSWSE<br>RCAIRYYSSKQAIFTQKDLPMKSEADLGGR<br>YSVSVMSSYERSKQCMKSVHSSIGNRLRSS<br>MSWTSSKVSNSVCRTINYLVPDVFKFMNVL<br>VCISLLIKMTAEANHIVTTQRRLKLDVEETE<br>RRKIEWELAFHHAILTQSAGQHPTIDEFRAY<br>IADKAPHLSEHIEPEEKAVVHQ	GTEWEDTHGSSNIDNPQWCIKRLIKGVYKP<br>KQLKEDMLANPFLPLYALLSPGVILAFYNS<br>GSLEYLMNHYIRVDSNVAVLLVVLKSLAK<br>KVSTSQSVLAQLQIIERSLPELIEAKANVNGP<br>DDAATRACNRFMGMLLHMAEPNWELADG<br>GYTILRDHSISILEKSYLQILDEAWNELSWSE<br>RCAIRYYSSKQAIFTQKDLPMKSEADLGGR<br>YSVSVMSSYERSKQCMKSVHSSIGNRLRSS<br>MSWTSSKVSNSVCRTINYLVPDVFKFMNVL<br>VCISLLIKMTAEANHIVTTQRRLKLDVEETE<br>RRKIEWELAFHHAILTQSAGQHPTIDEFRAY<br>IADKAPHLSEHIEPEEHHHHHHDYKDHDGD<br>YKDHDIDYKDDDDKKAVVHQ
6k1	AKRQSEQELERIIAFVALVLMMFDAERS<br>DCVTKILNKLKGLVATVEPTVYHQ	HHHHHHDYKDHDGDYKDHDIDYKDDDDK<br>AKRQSEQELERIIAFVALVLMMFDAERSDC<br>VTKILNKLKGLVATVEPTVYHQ	AKRQSEQELERIIAFVALVLMMFDAERSDC<br>VTKILNKLKGLVATVEHHHHHHDYKDHDG<br>DYKDHDIDYKDDDDKPTVYHQ
Cl	TLNDIEDDLSERNLFVDFELSSDGDMLQ<br>QLPAEKTFASWWSHQLSRGFTIPHYRTE<br>GKFMTFTRATATEVAGKIAHESDKDILL<br>MGAVGSGKSTGLPYHLSRKGNVLLLEP<br>TRPLAENVHKQLSQAPFHQNTTLRMRG<br>LTAFGSAPISVMTSGFALNYFANNRMRI<br>EEFDFVIFDECHVHDANAMAMRCLLHE<br>CDYSGKIIKVSATPPGREVEFSTQYPVSI<br>STEDTLSFQDFVNAQGSGSNCDVISKGD<br>NILVYVASYNEVDALSKLLIERDFKVTK<br>VDGRTMKVGNIEITTSGTPSKKHFIVAT<br>NIIENGVTLDIDVVADFGTKVLPYLDTD<br>SRMLSTTKTSINYGERIQRLGRVGRHKP<br>GHALRIGHTEKGLSEVPSCIATEAALKC	HHHHHHDYKDHDGDYKDHDIDYKDDDDK<br>TLNDIEDDLSERNLFVDFELSSDGDMLQQLP<br>AEKTFASWWSHQLSRGFTIPHYRTEGKFMT<br>FTRATATEVAGKIAHESDKDILLMGAVGSG<br>KSTGLPYHLSRKGNVLLLEPTRPLAENVHK<br>QLSQAPFHQNTTLRMRGLTAFGSAPISVMT<br>SGFALNYFANNRMRIEEFDFVIFDECHVHD<br>ANAMAMRCLLHECDYSGKIIKVSATPPGRE<br>VEFSTQYPVSISTEDTLSFQDFVNAQGSGSN<br>CDVISKGDNILVYVASYNEVDALSKLLIERD<br>FKVTKVDGRTMKVGNIEITTSGTPSKKHFIV<br>ATNIIENGVTLDIDVVADFGTKVLPYLDTDS<br>RMLSTTKTSINYGERIQRLGRVGRHKPGHA<br>LRIGHTEKGLSEVPSCIATEAALKCFTYGLP	TLNDIEDDLSERNLFVDFELSSDGDMLQQLP<br>AEKTFASWWSHQLSRGFTIPHYRTEGKFMT<br>FTRATATEVAGKIAHESDKDILLMGAVGSG<br>KSTGLPYHLSRKGNVLLLEPTRPLAENVHK<br>QLSQAPFHQNTTLRMRGLTAFGSAPISVMT<br>SGFALNYFANNRMRIEEFDFVIFDECHVHD<br>ANAMAMRCLLHECDYSGKIIKVSATPPGRE<br>VEFSTQYPVSISTEDTLSFQDFVNAQGSGSN<br>CDVISKGDNILVYVASYNEVDALSKLLIERD<br>FKVTKVDGRTMKVGNIEITTSGTPSKKHFIV<br>ATNIIENGVTLDIDVVADFGTKVLPYLDTDS<br>RMLSTTKTSINYGERIQRLGRVGRHKPGHA<br>LRIGHTEKGLSEVPSCIATEAALKCFTYGLP<br>VITNNVSTSILGNVTVKQARTMSVFEITPFY

	FTYGLPVITNNVSTSILGNVTVKQARTM<br>SVFEITPFYTSQVVRYDGSMHPQVHALL<br>KRFKLRDSEIVLNKLAIPHRGVNAWLTA<br>SEYARLGANVEDRRDVRIPFMCRDIPEK<br>LHLDMWDVIVKFKGDAGFGRLSSASAS<br>KVAYTLQTDVNSIQRTVTIIDTLIAEERR<br>KQEYFKTVTSNCVSSSNFSLQSITNAIKS<br>RMMKDHTCENISVLEGAKSQLLEFRNL<br>NADHSFATKTDGISRHFMSEYGALEAV<br>HHQ	VITNNVSTSILGNVTVKQARTMSVFEITPFY<br>TSQVVRYDGSMHPQVHALLKRFKLRDSEIV<br>LNKLAIPHRGVNAWLTASEYARLGANVED<br>RRDVRIPFMCRDIPEKLHLDMWDVIVKFKG<br>DAGFGRLSSASASKVAYTLQTDVNSIQRTV<br>TIIDTLIAEERRKQEYFKTVTSNCVSSSNFSL<br>QSITNAIKSRMMKDHTCENISVLEGAKSQLL<br>EFRNLNADHSFATKTDGISRHFMSEYGALE<br>AVHHQ	TSQVVRYDGSMHPQVHALLKRFKLRDSEIV<br>LNKLAIPHRGVNAWLTASEYARLGANVED<br>RRDVRIPFMCRDIPEKLHLDMWDVIVKFKG<br>DAGFGRLSSASASKVAYTLQTDVNSIQRTV<br>TIIDTLIAEERRKQEYFKTVTSNCVSSSNFSL<br>QSITNAIKSRMMKDHTCENISVLEGAKSQLL<br>EFRNLNADHSFATKTDGISRHFMSEYGALH<br>HHHHHDYKDHDGDYKDHDIDYKDDDDKE<br>AVHHQ
6k2	NTSDMSKFLKLKGKWNKTLITRDVLVL<br>CGVLGGGLWMVIQHLRSKMSEPVTHE	HHHHHHDYKDHDGDYKDHDIDYKDDDDK<br>NTSDMSKFLKLKGKWNKTLITRDVLVLCG<br>VLGGGLWMVIQHLRSKMSEPVTHE	NTSDMSKFLKLKGKWNKTLITRDVLVLCG<br>VLGGGLWMVIQHLRSKMSHHHHHHDYKD<br>HDGDYKDHDIDYKDDDDKEPVTHE
VPg	AKGKRQRQKLKFRNARDNKMGREVYG<br>DDDTIEHFFGDAYTKKGKSKGRTRGIG<br>HKNRKFINMYGFDPEDFSAVRFVDPLT<br>GATLDDNPLTDITLVQEHFGNIRMDLLG<br>EDELDSNEIRVNKTIQAYYMNNKTGKA<br>LKVDLTPHIPLKVCDLHATIAGFPEREN<br>ELRQTGKAQPINIDEVPRANNELVPVDH<br>E	HHHHHHDYKDHDGDYKDHDIDYKDDDDK<br>AKGKRQRQKLKFRNARDNKMGREVYGDD<br>DTIEHFFGDAYTKKGKSKGRTRGIGHKNRK<br>FINMYGFDPEDFSAVRFVDPLTGATLDDNP<br>LTDITLVQEHFGNIRMDLLGEDELDSNEIRV<br>NKTIQAYYMNNKTGKALKVDLTPHIPLKVC<br>DLHATIAGFPERENELRQTGKAQPINIDEVP<br>RANNELVPVDHE	AKGKRQRQKLKFRNARDNKMGREVYGDD<br>DTIEHFFGDAYTKKGKSKGRTRGIGHKNRK<br>FINMYGFDPEDFSAVRFVDPLTGATLDDNP<br>LTDITLVQEHFGNIRMDLLGEDELDSNEIRV<br>NKTIQAYYMNNKTGKALKVDLTPHIPLKVC<br>DLHATIAGFPERENELRQTGKAQPINIDEVP<br>RANNELHHHHHHDYKDHDGDYKDHDIDY<br>KDDDDKVPVDHE
NIa-Pro	SNSMFRGLRDYNPISNNICHLTNVSDGA<br>SNSLYGVGFGPLLLTNRHLFERNNGELV<br>IKSRHGEFVIKNTTQLHLLPIPDRDLLLIR<br>LPKDVPPFPQKLGFRQPEKGERICMVGS<br>NFQTKSITSIVSETSTIMPVENSQFWKH<br>WISTKDGQCGSPMVSTKDGKILGLHSL<br>ANFQNSINYFAAFPDDFAEKYLHTIEAH<br>EWVKHWKYNTSAISWGSLNIQASQPSG<br>LFKVSKLISDLDSTAVYAQ	HHHHHHDYKDHDGDYKDHDIDYKDDDDK<br>SNSMFRGLRDYNPISNNICHLTNVSDGASNS<br>LYGVGFGPLLLTNRHLFERNNGELVIKSRH<br>GEFVIKNTTQLHLLPIPDRDLLLIRLPKDVPP<br>FPQKLGFRQPEKGERICMVGSNFQTKSITSI<br>VSETSTIMPVENSQFWKHWISTKDGQCGSP<br>MVSTKDGKILGLHSLANFQNSINYFAAFPD<br>DFAEKYLHTIEAHEWVKHWKYNTSAISWG<br>SLNIQASQPSGLFKVSKLISDLDSTAVYAQ	SNSMFRGLRDYNPISNNICHLTNVSDGASNS<br>LYGVGFGPLLLTNRHLFERNNGELVIKSRH<br>GEFVIKNTTQLHLLPIPDRDLLLIRLPKDVPP<br>FPQKLGFRQPEKGERICMVGSNFQTKSITSI<br>VSETSTIMPVENSQFWKHWISTKDGQCGSP<br>MVSTKDGKILGLHSLANFQNSINYFAAFPD<br>DFAEKYLHTIEAHEWVKHWKYNTSAISWG<br>SLNIQASQPSGLFKVSKLISDLDSHHHHHHD<br>YKDHDGDYKDHDIDYKDDDDKTAVYAQ
NIb	TQQNRWMFEQLNGNLKAIAHCPSQLVT<br>KHTVKGKCQMFDLYLKLHDEAREYFQ<br>PMLGQYQKSKLNREAYAKDLLKYATPI<br>EAGNIDCDLFEKTVEIVVSDLRGYGFET<br>CNYVTDENDIFEALNMKSAVGALYKGK<br>KKDYFAEFTPEMKEEILKQSCERLFLGK<br>MGVWNGSLKAELRPLEKVEANKTRTFT<br>AAPLDTLLGGKVCVDDFNNQFYDHNLR<br>APWSVGMTKFYCGWDRLLESLPDGWV<br>YCDADGSQFDSSLSPYLINAVLNIRLGF<br>MEEWDIGEVMLRNLYTEIVYTPISTPDG<br>TLVKKFKGNNSGQPSTVVDNTLMVILA<br>VNYSLKKSGIPSELRDSIIRFFVNGDDLL<br>LSVHPEYEYILDTMADNFRELGLKYTFD<br>SRTREKGDLWFMSHQGHKREGIWIPKL<br>EPERIVSILEWDRSKEPCHRLEAICAAMI<br>ESWGYDKLTHEIRKFYAWMIEQAPFSSL<br>AQEGKAPYIAETALRKLYLDKEPAQED<br>LTHYLQAIFEDYEDGAEACVYHQ	THHHHHHDYKDHDGDYKDHDIDYKDDDD<br>KQQNRWMFEQLNGNLKAIAHCPSQLVTKH<br>TVKGKCQMFDLYLKLHDEAREYFQPMLGQ<br>YQKSKLNREAYAKDLLKYATPIEAGNIDCD<br>LFEKTVEIVVSDLRGYGFETCNYVTDENDIF<br>EALNMKSAVGALYKGKKKDYFAEFTPEMK<br>EEILKQSCERLFLGKMGVWNGSLKAELRPL<br>EKVEANKTRTFTAAPLDTLLGGKVCVDDF<br>NNQFYDHNLRAPWSVGMTKFYCGWDRLL<br>ESLPDGWVYCDADGSQFDSSLSPYLINAVL<br>NIRLGFMEEWDIGEVMLRNLYTEIVYTPIST<br>PDGTLVKKFKGNNSGQPSTVVDNTLMVILA<br>VNYSLKKSGIPSELRDSIIRFFVNGDDLLLSV<br>HPEYEYILDTMADNFRELGLKYTFDSRTRE<br>KGDLWFMSHQGHKREGIWIPKLEPERIVSIL<br>EWDRSKEPCHRLEAICAAMIESWGYDKLTH<br>EIRKFYAWMIEQAPFSSLAQEGKAPYIAETA<br>LRKLYLDKEPAQEDLTHYLQAIFEDYEDGA<br>EACVYHQ	TQQNRWMFEQLNGNLKAIAHCPSQLVTKH<br>TVKGKCQMFDLYLKLHDEAREYFQPMLGQ<br>YQKSKLNREAYAKDLLKYATPIEAGNIDCD<br>LFEKTVEIVVSDLRGYGFETCNYVTDENDIF<br>EALNMKSAVGALYKGKKKDYFAEFTPEMK<br>EEILKQSCERLFLGKMGVWNGSLKAELRPL<br>EKVEANKTRTFTAAPLDTLLGGKVCVDDF<br>NNQFYDHNLRAPWSVGMTKFYCGWDRLL<br>ESLPDGWVYCDADGSQFDSSLSPYLINAVL<br>NIRLGFMEEWDIGEVMLRNLYTEIVYTPIST<br>PDGTLVKKFKGNNSGQPSTVVDNTLMVILA<br>VNYSLKKSGIPSELRDSIIRFFVNGDDLLLSV<br>HPEYEYILDTMADNFRELGLKYTFDSRTRE<br>KGDLWFMSHQGHKREGIWIPKLEPERIVSIL<br>EWDRSKEPCHRLEAICAAMIESWGYDKLTH<br>EIRKFYAWMIEQAPFSSLAQEGKAPYIAETA<br>LRKLYLDKEPAQEDLTHYLQAIFEDYEDGA<br>EAHHHHHHDYKDHDGDYKDHDIDYKDDD<br>DKCVYHQ
CP	AGETLDAGLTDEQKQAEKEKKEREKAE<br>KERERQKQLALKKGKDVAQEEGKRDK<br>EVNAGTSGTFSVPRLKSLTSKMRVPRYE<br>KRVALNLDHLILYTPEQTDLSNTRSTRK<br>QFDTWFEGVMADYELTEQKMQIILNGL<br>MVWCIENGTSPNINGMWVMMDGDDQ<br>VEFPIKPLIDHAKPTFRQIMAHFSDVAEA<br>YIEKRNQDRPYMPRYGLQRNLTDMSLA<br>RYAFDFYEMTSRTPIRAREAHIQMKAA<br>ALRGANNNLFGLDGNVGTTVENTERHT<br>TEDVNRNMHNLLGVQGL	AGETLDAGHHHHHHDYKDHDGDYKDHDI<br>DYKDDDDKLTDEQKQAEKEKKEREKAEKE<br>RERQKQLALKKGKDVAQEEGKRDKEVNA<br>GTSGTFSVPRLKSLTSKMRVPRYEKRVALN<br>LDHLILYTPEQTDLSNTRSTRKQFDTWFEG<br>VMADYELTEQKMQIILNGLMVWCIENGTSP<br>NINGMWVMMDGDDQVEFPIKPLIDHAKPT<br>FRQIMAHFSDVAEAYIEKRNQDRPYMPRYG<br>LQRNLTDMSLARYAFDFYEMTSRTPIRARE<br>AHIQMKAAALRGANNNLFGLDGNVGTTVE<br>NTERHTTEDVNRNMHNLLGVQGL	AGETLDAGLTDEQKQAEKEKKEREKAEKE<br>RERQKQLALKKGKDVAQEEGKRDKEVNA<br>GTSGTFSVPRLKSLTSKMRVPRYEKRVALN<br>LDHLILYTPEQTDLSNTRSTRKQFDTWFEG<br>VMADYELTEQKMQIILNGLMVWCIENGTSP<br>NINGMWVMMDGDDQVEFPIKPLIDHAKPT<br>FRQIMAHFSDVAEAYIEKRNQDRPYMPRYG<br>LQRNLTDMSLARYAFDFYEMTSRTPIRARE<br>AHIQMKAAALRGANNNLFGLDGNVGTTVE<br>NTERHTTEDVNRNMHNLLGVQGLHHHHH<br>HDYKDHDGDYKDHDIDYKDDDDK
