# Supplementary material for: Contrasting Effects of Tagging Turnip Mosaic Virus Proteins
Source: Pathogens. 2026 Jun 8;15(6):611. doi: 10.3390/pathogens15060611 (PMC13305232; doi:10.3390/pathogens15060611)
Supplement: Supplementary file 1 [file pathogens-15-00611-s001.zip › Table_S5_SASA_2.pdf]

Table S5. Solvent-accessible surface area (SASA) analysis of the predicted structure of other potyviral proteins with 6xHis-3xFLAG tags at N- and C-termini. Bold fonts indicate a notable (>1%) predicted tag interference with the protein structure. NA indicates the data are not available due to the lack of a well-defined substrate-binding pore.

**Active.site[a]**	**Wotail.AS[b]**	**AS_5A[c]**	**Wotail.AS5A[d]**	**Pore.lining[e]**	**Wotail.pore[f]**	**%diff.AS[g]**	**%diff.AS_5A[h]**	**%diff.pore.lining[i]**	**Active.site[a]**
P1_N	-2.4	-2.4	1283.2	1283.2	NA	NA	0	0	NA
P1_C	59.9	81.8	1288.9	1413.3	NA	NA	-26.8	-8.8	NA
P3_N	28723.9	28808.9	28723.9	28808.9	NA	NA	-0.3	-0.3	NA
P3_C	27671.6	27729.4	27671.6	27729.4	NA	NA	-0.2	-0.2	NA
6K1_N	2217.6	2225.4	3528.6	3660.8	NA	NA	-0.3	-3.6	NA
6K1_C	2174.8	2174.8	3622.4	3670.7	NA	NA	0	-1.3	NA
CI_N	35830.5	35885.6	38328.7	38416.3	NA	NA	-0.2	-0.2	NA
CI_C	35793.8	35863.8	38110.1	38180.1	NA	NA	-0.2	-0.2	NA
6K2_N	2517.8	2517.8	3856.7	3856.7	NA	NA	0	0	NA
6K2_C	2386.9	2451.6	3700.6	3840.3	NA	NA	-2.6	-3.6	NA
VPg_N	1240.5	1240.5	2437	2437	NA	NA	0	0	NA
VPg_C	1302.1	1302.1	2402.7	2404.6	NA	NA	0	-0.1	NA
NIa_N	12.1	12.1	379.3	379.3	469.7	469.7	0	0	0
NIa_C	8.4	49.1	323.6	443	411.7	505.2	-82.8	-26.9	-18.5
CP_N	211.1	211.1	1021.9	1062.6	NA	NA	0	-3.8	NA
CP_C	471.3	471.3	1544.9	1544.9	NA	NA	0	0	NA

[a] Values for residues that form the active site.

[b] Without tail Active.site.

[c] Residues proximal to the active site without tail.

[d] Residues proximal to the active site.

[e] Values for residues that line the pore.

[f] Values for residues that line the pore without tail.

[g] Percent change in the active site.

[h] Percent change in residues proximal to the active site.

[i] Percent change in the pore-lining region.
